# DeSUMOylation of chromatin-bound proteins limits the rapid transcriptional reprogramming induced by daunorubicin in acute myeloid leukemias

**DOI:** 10.1093/nar/gkad581

**Published:** 2023-07-18

**Authors:** Mathias Boulanger, Mays Aqrouq, Denis Tempé, Chamseddine Kifagi, Marko Ristic, Dana Akl, Rawan Hallal, Aude Carusi, Ludovic Gabellier, Marion de Toledo, Jon-Otti Sigurdsson, Tony Kaoma, Charlotte Andrieu-Soler, Thierry Forné, Eric Soler, Yosr Hicheri, Elise Gueret, Laurent Vallar, Jesper V Olsen, Guillaume Cartron, Marc Piechaczyk, Guillaume Bossis

**Affiliations:** IGMM, Univ. Montpellier, CNRS, Montpellier, France; IGMM, Univ. Montpellier, CNRS, Montpellier, France; IGMM, Univ. Montpellier, CNRS, Montpellier, France; IGMM, Univ. Montpellier, CNRS, Montpellier, France; IGMM, Univ. Montpellier, CNRS, Montpellier, France; IGMM, Univ. Montpellier, CNRS, Montpellier, France; IGMM, Univ. Montpellier, CNRS, Montpellier, France; IGMM, Univ. Montpellier, CNRS, Montpellier, France; IGMM, Univ. Montpellier, CNRS, Montpellier, France; Service d’Hématologie Clinique, CHU de Montpellier, 80 Avenue Augustin Fliche, 34091 Montpellier, France; IGMM, Univ. Montpellier, CNRS, Montpellier, France; Proteomics Program, Novo Nordisk Foundation Center For Protein Research, Faculty of Health and Medical Sciences, University of Copenhagen, Blegdamsvej 3B, DK-2200 Copenhagen, Denmark; Genomics Research Unit, Luxembourg Institute of Health, 84, Val Fleuri, L-1526 Luxembourg, Luxembourg; IGMM, Univ. Montpellier, CNRS, Montpellier, France; Université de Paris, Laboratory of Excellence GR-Ex, Paris, France; IGMM, Univ. Montpellier, CNRS, Montpellier, France; IGMM, Univ. Montpellier, CNRS, Montpellier, France; Université de Paris, Laboratory of Excellence GR-Ex, Paris, France; Service d’Hématologie Clinique, CHU de Montpellier, 80 Avenue Augustin Fliche, 34091 Montpellier, France; MGX-Montpellier GenomiX, Univ. Montpellier, CNRS, INSERM, Montpellier, France; Genomics Research Unit, Luxembourg Institute of Health, 84, Val Fleuri, L-1526 Luxembourg, Luxembourg; Proteomics Program, Novo Nordisk Foundation Center For Protein Research, Faculty of Health and Medical Sciences, University of Copenhagen, Blegdamsvej 3B, DK-2200 Copenhagen, Denmark; IGMM, Univ. Montpellier, CNRS, Montpellier, France; Service d’Hématologie Clinique, CHU de Montpellier, 80 Avenue Augustin Fliche, 34091 Montpellier, France; IGMM, Univ. Montpellier, CNRS, Montpellier, France; IGMM, Univ. Montpellier, CNRS, Montpellier, France

## Abstract

Genotoxicants have been used for decades as front-line therapies against cancer on the basis of their DNA-damaging actions. However, some of their non-DNA-damaging effects are also instrumental for killing dividing cells. We report here that the anthracycline Daunorubicin (DNR), one of the main drugs used to treat Acute Myeloid Leukemia (AML), induces rapid (3 h) and broad transcriptional changes in AML cells. The regulated genes are particularly enriched in genes controlling cell proliferation and death, as well as inflammation and immunity. These transcriptional changes are preceded by DNR-dependent *de*SUMOylation of chromatin proteins, in particular at active promoters and enhancers. Surprisingly, inhibition of SUMOylation with ML-792 (SUMO E1 inhibitor), dampens DNR-induced transcriptional reprogramming. Quantitative proteomics shows that the proteins *de*SUMOylated in response to DNR are mostly transcription factors, transcriptional co-regulators and chromatin organizers. Among them, the CCCTC-binding factor CTCF is highly enriched at SUMO-binding sites found in *cis*-regulatory regions. This is notably the case at the promoter of the DNR-induced *NFKB2* gene. DNR leads to a reconfiguration of chromatin loops engaging CTCF- and SUMO-bound *NFKB2* promoter with a distal *cis*-regulatory region and inhibition of SUMOylation with ML-792 prevents these changes.

## Graphical Abstract

**Graphical Abstract:**
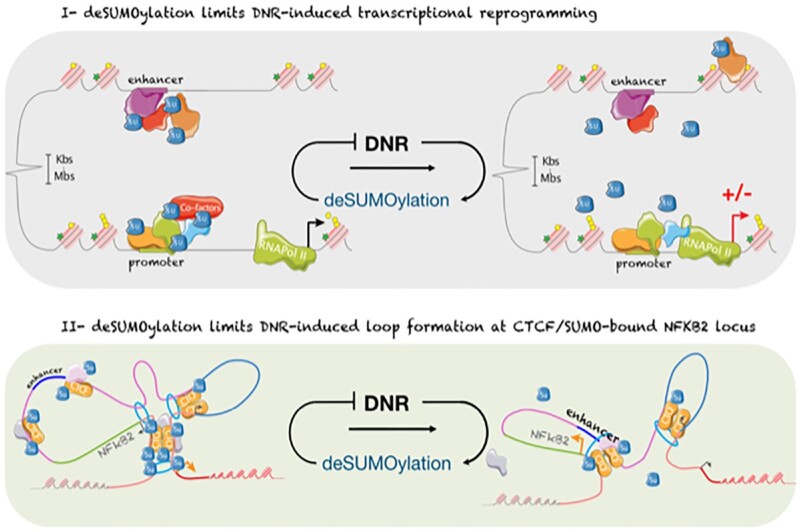

## INTRODUCTION

Acute myeloid leukemias (AML) are severe hematological malignancies, which arise through the acquisition of oncogenic mutations by hematopoietic stem or progenitor cells from the myeloid lineage. Although AML constitutes a highly heterogenous group of diseases, most of them are treated similarly with the combination of one anthracycline, such as Daunorubicin (DNR) or Idarubicin (IDA), and the nucleoside analogue Cytarabine (Ara-C) (1–3). Most patients respond to this treatment. However, a large proportion of them relapse and become refractory to the drugs, which contributes to the dismal prognosis of this disease (2,3). It is therefore critical to better understand the mode(s) of action of these drugs to find ways to overcome chemoresistance.

The DNA-damaging properties of both Ara-C and DNR are essential for therapeutical efficacy and have been characterized extensively (4,5). However, these drugs also display many other cellular effects that can both favor or counteract their ability to induce cell death. For example, anthracyclines can induce fast production of reactive oxygen species (ROS) that contribute to apoptosis induction by activating various signaling pathways (6). On the other hand, Ara-C and DNR also activate, at the same time, many pro-survival pathways that mitigate their pro-apoptotic actions. This is notable for the PI3K/AKT (7), MAPK (8) and NF-κB (9,10) pathways, as their inhibitions potentiate genotoxics-induced cell death in cancer cells. Finally, both anthracyclines and Ara-C have long been known to alter transcriptional programs on the mid/long term (day-range) when used at sublethal doses (11,12). However, how Ara-C and DNR contribute to gene expression changes at early times after the start of a treatment, has been poorly investigated.

We have formerly shown that one early consequence of DNR and Ara-C treatments is ROS-dependent *de*SUMOylation of proteins in chemosensitive AMLs, which participates in induction of apoptosis (13). SUMOylation consists of reversible, covalent modification of proteins by the ubiquitin-related peptidic post-translational modifiers SUMO-1 to -3. SUMO-1 is 50% identical to SUMO-2 and -3, which are 95% identical and frequently referred to as SUMO-2/3 as their individual functions can often not be distinguished. The three SUMOs are conjugated by a conserved enzymatic cascade comprising one SUMO-activating enzyme (SAE1/SAE2 dimer; also called SUMO E1), one SUMO-conjugating enzyme (Ubc9; also called SUMO E2) and several SUMO E3s that facilitate SUMO transfer from the E2 onto its protein targets. SUMOylation is highly dynamic thanks to various isopeptidases (also called *de*SUMOylases) that remove SUMO from its substrates (14). Thousands of SUMOylated proteins involved in many cellular processes have now been identified (15). However, one of the main biological processes associated with SUMOylation is the control of gene expression. Numerous transcription factors and co-regulators, as well as histones and the basal transcription machinery are SUMOylated (16). Moreover, genome-wide studies have revealed that SUMOylated proteins are highly enriched at gene regulatory regions, including promoters and enhancers (17–21). Their SUMOylation is likely to occur on chromatin as both SUMO conjugating (E1, E2 and E3s) and deconjugating enzymes can bind to the chromatin (17,22–24). Although SUMOylation of chromatin-bound proteins has often been associated with gene silencing or gene expression limitation (17,24–26), it can also participate in the activation of certain genes such as ribosomal genes (19,20), fibroblastic genes in mouse embryonic fibroblasts (MEFs)(25), PPARg/RXR target genes during adipocyte differentiation (21) as well as RNA-polymerase III (27) controlled genes. Overall, the impact of SUMOylation on transcription appears to be dependent on both genes and signaling contexts, as well as on the nature of the conjugated proteins and of the chromatin environment (16).

To better understand the complex mode of action of these drugs, we explored the early effects of Ara-C and DNR on gene expression in AML cells, together with the contribution of SUMOylation to transcriptome reprogramming. We report that DNR induces rapid and broad gene expression changes that are preceded by *de*SUMOylation of chromatin-bound proteins, in particular at active promoters and enhancers, whereas the effect of Ara-C is much more limited. Intriguingly, we found that inhibition of SUMOylation limits DNR-induced changes in gene expression. Among the proteins most rapidly *de*SUMOylated in response to DNR, we identified the CTCF insulator protein, which was found highly enriched in regions of the genome marked by SUMO. This notably concerns the *NFKB2* gene, whose DNR-induced expression is preceded by rearrangement of chromatin loops involving its SUMO/CTCF-marked promoter and *cis*-regulatory elements.

## MATERIALS AND METHODS

### Pharmacologic inhibitors, reagents and antibodies

Cytosine-β-D-arabinofuranoside (Ara-C), daunorubicin-hydrochloride (DNR), boric acid, protein-G beads, SILAC medium, dimetyl-pimelidade (DMP) were from Sigma. Dialysed serum for SILAC experiments was from Eurobio Abcys. ML-792 and TAK-981 were provided Takeda Development Center Americas. Lysine and arginine isotopes were from Cambridge Isotope Laboratories. Anti-SUMO-1- (21C7), SUMO2- (8A2) and control- (anti-BrdU, G3G4) hybridomas were obtained from the Developmental Studies Hybridoma Bank (DSHB). The goat polyclonal anti-SUMO-2/3 antibody was described previously (28). The anti-CTCF antibody was from Diagenode (C15410210) for immunoblotting and from Millipore (07-729) for CUT&RUN. The NFkB2 antibody was from Millipore (06-413).

### Cell culture and genotoxic treatment

HL-60 cells were obtained from the ATCC, authenticated by LGC and regularly tested for the absence of mycoplasma. They were cultured at 37°C in the presence of 5% CO_2_ in RPMI (Eurobio) medium supplemented with 10% decomplemented (30 min at 56°C) fetal bovine serum (FBS) and penicillin and streptomycin. After thawing, cells were split at 0.3 × 10^6^/ml every 2 to 3 days for no more than 10 passages. HEK293T cells were cultured at 37°C in the presence of 5% CO_2_ in DMEM (Eurobio) medium supplemented with 10% decomplemented FBS and penicillin and streptomycin. HL-60 cells were seeded at 0.3 × 10^6^/ml the day before treatment with drugs at 1 μM for DNR and 2 μM for Ara-C. Cells were treated for 2 h for ChIP-Seq, 4C and CUT&RUN experiments and 3 h for Affimetrix transcriptomic and RNA-Seq. For SILAC experiments, HL-60 cells were grown in SILAC medium supplemented with dialyzed serum and K0/R0 (light condition), K4/R6 (medium condition), K8/R10 (heavy condition) amino acid isotopes for 21 days until incorporation of amino acids isotopes reached 99%, as measured by mass spectrometry. SILAC labelled cells were then treated or not with 1 μM DNR for 2 h. Hybridomas were grown in CellLine bioreactors (Integra) according to the manufacturer's protocol using RPMI in the cell compartment and RPMI + 10% FCS in the medium compartment. Antibodies were harvested from the cell compartment after 7 days of culture.

### AML patients' cells and healthy donors PBMCs

Bone marrow aspirates or blood were collected after obtaining written informed consent from patients under the frame of the Declaration of Helsinki and after approval by the Institutional Review Board (Ethical Committee ‘Sud Méditerranée 1,’ ref 2013-A00260-45, HemoDiag collection). Healthy donor leukocytes were collected from blood donors of the Montpellier Etablissement Français du Sang. Fresh leukocytes were purified as previously described (29) using density-based centrifugation using Histopaque 1077 from Sigma and directly lysed for RNA preparation or frozen and stored in liquid nitrogen.

### Gene silencing

The PLK0 lentivirus expressing scramble (SHC002) and UBC9 (NM_003345.3–545S1C1) shRNA expressing vectors were from Sigma. Viral particles were produced and used to transduce HL60 cells as described previously (30). Cells were selected with puromycin (1 μg/ml) for 3 weeks.

### Microarray-based whole transcript expression analysis and profiling

Total RNAs were extracted using the GenEluteTM Mammalian Total RNA kit (Sigma) and treated with DNAse I according to the manufacturer's specifications. For each condition, three independent batches of RNA were prepared and controlled for purity and integrity using the Agilent 2100 Bioanalyzer with RNA 6000 Nano LabChip kits (Agilent Technologies). Only RNA with no sign of contamination or degradation (RIN > 9) were processed to generate amplified and biotinylated sense-strand cDNA targets using the GeneChip® WT PLUS Reagent kit from Affymetrix according to the manufacturer's specifications. After fragmentation, cDNA targets were used to probe Affymetrix GeneChip® Human Gene 2.0 ST arrays, which were then washed, stained and scanned according to Affymetrix instructions (manual P/N 702731 Rev.3).

### Microarray data analysis

CEL files generated after array scanning were imported into the Partek® Genomics Suite 6.6 (Partek Inc.) for estimating transcript cluster expression levels from raw probe signal intensities using default Partek settings. Resulting expression data were then imported into R (http://www.R-project.org/) for further analysis. First, non-specific filtering was applied to remove transcript clusters with no specified chromosome location. Then, boxplots, density plots, relative log expressions (RLE) and sample pairwise correlations were generated to assess the quality of the data. They revealed no outlier within the series of hybridizations. Principal component analysis (PCA) was also applied to the dataset. The first two components of the PCA could separate samples according to the treatment. Thus, the treatment was considered as the unique source of variability. Finally, the LIMMA package (31) was used to detect differentially expressed genes (DEG) between treated and non-treated samples. A linear model with treatment as unique factor was fitted to the data before applying eBayes function to calculate the significance of the difference in gene expression between the two groups. *P*-values were adjusted by Benjamin and Hochberg's False Discovery Rate (FDR) and genes with FDR <0.05 and absolute linear Fold Change (FC) greater or equals to 2 were considered as DEG. Microarray data are available at ArrayExpress under the accession number E-MATB-4895.

### RT-qPCR assays

Total mRNAs were purified using the GenElute Mammalian Total RNA kit (Sigma-Aldrich). After 1 h of DNase I (4U, NEB) treatment in the presence of RNasin (2.5U; Promega), 1 μg of total RNA was used for cDNA synthesis using the Maxima First Strand cDNA kit (Thermo Fisher Scientific). qPCR assays were conducted using Taq platinum (Invitrogen) and the LightCycler 480 device (Roche) with specific DNA primers (Table 1). Data were normalized to the mRNA levels of the housekeeping genes TBP and S26 or GAPDH.

**Table 1.** Sequences of the primers used for RT-PCR experiments

**Gene transcript name**	**Primer strand**	**Sequence**
FOSB	Forward	GGAGACGCTCACCCCAGAG
	Reverse	AGCTCTGCTTTTTCTTCCTCCA
CXCL10	Forward	CCACGTGTTGAGATCATTGCTAC
	Reverse	TCGATTTTGCTCCCCTCTGGT
NFκB2 isoform 1	Forward	GGATCCACGTCGACACCGTT
	Reverse	ACCATCCAGACCTGGGTTGTAG
NFκB2 all isoforms	Forward	GCAGGCCTTTGGGGACTTCT
	Reverse	TGCACCTCTTCCTTGTCTTCCA
UBC9	Forward	CCGTGGGAAGGAGGCTTGTT
	Reverse	TGGCCTCCAGTCCTTGTCC
IER3	Forward	CTCGAGTGGTCCGGCG
	Reverse	ACGATGGTGAGCAGCAGAAA
TBP	Forward	TTTTCTTGCTGCCAGTCTGGAC
	Reverse	CACGAACCACGGCACTGATT
S26	Forward	CTGCACTAACTGTGCCCGATGCGTG
	Reverse	GACGCTCGCTTCAGAAATGTCCCTG

### RNA-seq libraries preparation and sequencing

RNA-Seq were performed as described previously (32). Total RNAs were purified using the GenElute Mammalian Total RNA kit (Sigma-Aldrich), treated with DNase I (4U; New England Biolabs) in the presence of RNasin (2.5U; Promega) and re-purified. RNA quality was assessed using a BioAnalyzer Nano 6000 chip (Agilent). Three independent experiments were performed. Libraries were prepared using TruSeq®Stranded mRNA Sample Preparation kit (Illumina). Libraries were sequenced using an Illumina Hiseq 2500 sequencer as single-end 50-base reads. Image analysis and base calling were performed using HiSeq Control Software (HCS), Real-Time Analysis (RTA) and bcl2fastq.

### Preparation of DNA for ChIP-seq

A total of 18 × 10^6^ cells were cross-linked with 1% paraformaldehyde for 8 min. Paraformaldehyde was then neutralized with 125 mM glycine for 10 min. Cross-linked cells were washed with cold PBS, resuspended in a cell lysis buffer (PIPES 5 mM pH7.5, KCl 85 mM, NP40 0.5%, *N*-ethyl maleimide 20 mM, aprotinin, + pepstatin + leupeptin 1 μg/ml each, AEBSF 1 mM) and incubated at 4°C for 10 min. Nuclei were centrifuged (5000 rpm for 10 min at 4°C) and resuspended in a nucleus lysis buffer (Tris–HCl 50 mM pH 7.5, SDS 1%, EDTA 10 mM, *N*-ethyl maleimide 20 mM, aprotinin + pepstatin + leupeptin 1 μg/ml each, 1 mM AEBSF) and incubated at 4°C for 2.5 h. Lysates were then sonicated for 20 cycles of 30 s, each at 4°C, using the Bioruptor Pico (Diagenode). After sonication, samples were centrifuged (13 000 rpm at 4°C for 10 min) and the supernatants were diluted 100-fold in the immunoprecipitation buffer (Tris–HCl 50 mM pH 7.5, NaCl 167 mM, *N*-ethyl maleimide 5 mM, EDTA 1 mM, Triton X100 1.1%, SDS 0.01%, aprotinin + pepstatin + leupeptin 1 μg/ml each, AEBSF 1 mM) with 2 μg of antibodies and Dynabeads Protein G (Thermo Fisher Scientific). Control immunoprecipitation (IP) were performed using the G3G4 antibody (anti BrdU antibody). IPs were performed at 4°C overnight. Beads were then washed in low-salt buffer (Tris–HCl 50 mM pH 7.5, NaCl 150 mM, Triton X100 1%, SDS 0.1%, EDTA 1 mM), high-salt buffer (Tris–HCl 50 mM pH 7.5, NaCl 500 mM, Triton X100 1%, SDS 0.1%, EDTA 1 mM), LiCl salt (Tris–HCl 20 mM pH 7.5, LiCl 250 mM, NP40 1%, deoxycholic acid 1%, EDTA 1 mM), and TE buffer (Tris–HCl 10 mM pH7.5, Tween20 0.2%, EDTA 1 mM). Elution was done in 200 μl of NaHCO3 100 mM containing SDS 1%. Chromatin cross-linking was reversed by overnight incubation at 65°C with NaCl 280 mM followed by 1.5 h at 45°C with Tris–HCl 35 mM pH6.8, EDTA 9 mM containing 88 μg/ml of RNAse and 88 μg/ml of proteinase K. Immunoprecipitated DNAs were purified using the NucleoSpin Gel and PCR Clean-up Kit (Macherey-Nagel).

### ChIP-seq libraries preparation and sequencing

For SUMO-2/3 ChIP-seq, immunoprecipitated DNA and corresponding inputs from three independent experiments were pooled before library preparation and sequencing. After the analysis of DNA integrity and the DNA fragment size using the BioAnalyser DNA HS chip (Agilent), ChIP-seq libraries were prepared by the Montpellier MGX platform (https://www.mgx.cnrs.fr) using TruSeq®ChIP Sample Preparation kits (Illumina). The sequencing was processed on Hi-SEQ 2000 (Illumina) as single-end 50 base reads. Image analysis and base calling were performed using HCS and RTA. Demultiplexing was performed using Illumina's sequencing analysis software (CASAVA 1.8.2) and bcl2fastq.

### CUT&RUN preparation and sequencing

HL-60 cells were treated with 1 μM DNR and 0.5 μM ML-792 for 2 h (3 independent biological replicates). Cells (10e5/condition) were harvested by centrifugation, washed once with PBS and resuspended in 100 μl PBS. BioMag Plus Concanavalin A beads (12.5 μl slurry, Polysciences, catalog #86057) were activated in 100 μl of activation buffer (20 mM HEPES (pH7.5), 10 mM KCl, 1 mM CaCl_2_). Beads were washed with activation buffer twice and resuspended in 100 μl activation buffer. Cells were bound to beads by mixing 100 μl of activated beads with 100 μl cells in PBS and incubated at room temperature for 15 min with rotation. Cell-bead mixture was collected with a magnet and resuspended in 50 μl wash-digest buffer (20 mM HEPES pH7.5, 150 mM NaCl, 0.5 mM Spermidine, 0.1% Digitonin, 1X Protease Inhibitors (EDTA-free)). Antibodies were diluted 1:100 in 50 μl wash-digest buffer and added to each cell-bead slurry for overnight incubation at 4°C on a rotator. Cell-bead mixture was collected by magnet and resuspended in 95 μl wash-digest buffer/condition. pAG-MNase was then added for 2 h at room temperature on a rotator. Cell-bead mixture was collected by magnet and resuspended in 100 μl low salt buffer (20mM HEPES pH 7.5, 0.5mM spermidine, 0.1% Digitonin) and incubated for 5 min at room temperature. Cell-bead mixture was collected by magnet and resuspended in 100 μl incubation buffer (3.5 mM HEPES pH 7.5, 10 mM CaCl_2_, 0.1% Digitonin) followed by an incubation on ice for 30 min to activate the pAG-MNase. The digestion was halted by the addition of 100 μl 2X stop buffer/condition (20 mM HEPES pH7.5) 340 mM NaCl, 20 mM EDTA, 6 mM EGTA, 50 μg/ml RNase A, 0.1% Digitonin) and incubation for 20 min at 37°C. Supernatants were collected by magnet and DNA fragments were purified using Monarch^®^ PCR & DNA Cleanup Kit (catalog #T1030). Sequencing libraries were prepared using NEBNext® Ultra™ II DNA Library Prep Kit (Illumina, catalog #E7645) with fragments amplification for 15 cycles and purification using cleanNGS beads (CleanNA, catalog #CNGS-0001). Libraries were analyzed by capillary electrophoresis (Fragment Analyzer, NGS HS kit) and sequenced by Illumina Novaseq 6000 sequencer as paired-end 50 base reads.

### 4C-seq experiments

Chromatin for 4C-Seq experiments was prepared essentially as previously described (33,34). A total of 7 × 10^6^ cells in 10 ml of medium were cross-linked with formaldehyde 2% for 10 min at room temperature (RT). Formaldehyde was then neutralized with 125 mM glycine for 10 min at 4°C. After a wash with cold PBS, cells were resuspended in 5 ml of lysis buffer (Tris–HCl 10 mM pH 8, NaCl 10 mM, NP-40 0.2%, aprotinin + pepstatin + leupeptin 1 μg/ml each, AEBSF 1 mM) and incubated on ice for 20 min. Cells were pelleted 5 min at 380 g at 4°C, resuspended in 1 ml of lysis buffer and snap frozen in liquid nitrogen. Lysates were thawed at 37°C and centrifuged at 18000 g at RT for 5 min. Cell pellets were resuspended in 700 μl of first enzyme manufacturer buffer 1X (NlaIII – cutsmart [NEB – R0125L]) and homogenized on ice (50 strokes in total) with a 1 ml Dounce homogenizer. Cells were permeabilized using SDS 0.3%, at 37°C for 1 h under orbital shaking (1 krpm) on an Eppendorf thermomixer). SDS was displaced by adding TritonX100 1.65% and continuing orbital shaking at 37°C for 1 h. A 100 μl sample of the reaction mix was taken as a negative control for the first digestion. The digestion with NlaIII enzyme was performed at 37°C for 24 h under orbital shaking (1 krpm) using 3 sequential additions of 300 U of enzymes at regular intervals. Before enzyme inactivation at 65°C for 20 min, 100μl of the reaction mix was collected as a restriction enzyme digestion control. The ligation step was performed overnight at 16°C in 8 ml of a reaction mix adjusted to 1× of ligase reaction buffer and containing 800 μl of the restriction enzyme reaction mix, 240 U of T4 DNA ligase HC (Thermo scientific, EL0013) and ATP 0.04 mM. Proteinase K (300 μg) was added to ligated DNA products and the reaction was incubated an at 56°C for 1 h. Decrosslinking was achieved in an incubation step of 6 h at 65°C. The two control tubes also underwent the proteinase K and decrosslinking steps. Then, all samples were treated with 300 μg of RNAse at 37°C for 30 min. DNA purifications were performed using phenol:chloroform:Isoamyl alcohol 25:24:1 (PCI). DNAs were precipitated at −20°C overnight using 2 volumes of EtOH in the presence of NaCl 250 mM and 20 μg of glycogen (Thermo). DNAs were pelleted by centrifugation (10 krpm) at 4°C and washed using 70% EtOH. Pellets were dried at room temperature and resuspended in 50 μl of water. 10 μl samples were collected from both controls and ligated DNA products and electrophoresed through an agarose gel to control the digestion and ligation steps. Ligation products were digested at 37°C for 2.5 h under orbital shaking (1krpm) suing 100 U of the second restriction enzyme (DpnII from New England Biolabs, reference R0543M). The second restriction enzyme was inactivated and a second ligation was performed under the same condition as above. 4C libraries were purified with PCI and precipitated as described above. 4C libraries were amplified using specific primers composed of P5/P7 Illumina sequence supplemented with indexes and sequences corresponding to the *NFKB2* promoter (viewpoint) (Table 2). The ‘Expend Long Template PCR System’ kit (Roche) was used using 300 ng of the 4C library following the manufacturer's instruction. The following amplification parameters were used: denaturation for 2 min at 94°C followed by 30 cycles (94°C – 15 s, 58°C – 1 min and 68°C – 3 min) and 7 min at 68°C. 4C libraries were purified with the ‘Gel and PCR clean up’ kit from Macherey-Nagel using NTI solution diluted 6 times and an elution buffer pre-heated at 70°C. After 3 PCR amplification rounds, all 4C libraries for the same sample were pooled, purified and cleaned up using Agencourt AMPure XP beads (ratio 1:1) using EtOH 80% as a washing solution. The libraries were sequenced using the Illumina Hiseq 2500 sequencer as single-end 125 base reads following Illumina's instructions. Image analysis and base calling were performed using the HiSeq Control Software (HCS), Real-Time Analysis (RTA) and bcl2fastq.

**Table 2.** PCR amplification primer to capture NFKB2 promoter interacting regions

**Primer name**	**Condition**	**Sequence**
For-P5-illuminaSeq-**NFκB2-NlaIII**	ALL	AATGATACGGCGACCACCGAGATCTACACTCTTTCCCTACACGACGCTCTTCCGATCT**CGTGACGCACGGAAACGTC**
Rev-P7-indexT1-**NFκB2-DpnII**	DMSO rep 1	CAAGCAGAAGACGGCATACGAGATAACGTGATGTGACTGGAGTTCAGACGTGTGCTCTTCCGATC**GCCTAACGCTTGGCTTTCTC**
Rev-P7-indexT2-**NFκB2-DpnII**	DNR rep 1	CAAGCAGAAGACGGCATACGAGATAAACATCGGTGACTGGAGTTCAGACGTGTGCTCTTCCGATC**GCCTAACGCTTGGCTTTCTC**
Rev-P7-indexT3-**NFκB2-DpnII**	ML-792 rep 1	CAAGCAGAAGACGGCATACGAGATATGCCTAAGTGACTGGAGTTCAGACGTGTGCTCTTCCGATC**GCCTAACGCTTGGCTTTCTC**
Rev-P7-indexT4-**NFκB2-DpnII**	ML-792-DNR rep 1	CAAGCAGAAGACGGCATACGAGATAGTGGTCAGTGACTGGAGTTCAGACGTGTGCTCTTCCGATC**GCCTAACGCTTGGCTTTCTC**
Rev-P7-indexT5-**NFκB2-DpnII**	DMSO rep 2	CAAGCAGAAGACGGCATACGAGATACCACTGTGTGACTGGAGTTCAGACGTGTGCTCTTCCGATC**GCCTAACGCTTGGCTTTCTC**
Rev-P7-indexT6-**NFκB2-DpnII**	DNR rep 2	CAAGCAGAAGACGGCATACGAGATACATTGGCGTGACTGGAGTTCAGACGTGTGCTCTTCCGATC**GCCTAACGCTTGGCTTTCTC**
Rev-P7-indexT7-**NFκB2-DpnII**	ML-792 rep 2	CAAGCAGAAGACGGCATACGAGATCAGATCTGGTGACTGGAGTTCAGACGTGTGCTCTTCCGATC**GCCTAACGCTTGGCTTTCTC**
Rev-P7-indexT8-**NFκB2-DpnII**	ML-792-DNR rep 2	CAAGCAGAAGACGGCATACGAGATCATCAAGTGTGACTGGAGTTCAGACGTGTGCTCTTCCGATC**GCCTAACGCTTGGCTTTCTC**
Rev-P7-indexT9-**NFκB2-DpnII**	DMSO rep 3	CAAGCAGAAGACGGCATACGAGATCGCTGATCGTGACTGGAGTTCAGACGTGTGCTCTTCCGATC**GCCTAACGCTTGGCTTTCTC**
Rev-P7-indexT10-**NFκB2-DpnII**	DNR rep 3	CAAGCAGAAGACGGCATACGAGATACAAGCTAGTGACTGGAGTTCAGACGTGTGCTCTTCCGATC**GCCTAACGCTTGGCTTTCTC**
Rev-P7-indexT11-**NFκB2-DpnII**	ML-792 rep 3	CAAGCAGAAGACGGCATACGAGATCTGTAGCCGTGACTGGAGTTCAGACGTGTGCTCTTCCGATC**GCCTAACGCTTGGCTTTCTC**
Rev-P7-indexT12-**NFκB2-DpnII**	ML-792-DNR rep 3	CAAGCAGAAGACGGCATACGAGATAGTACAAGGTGACTGGAGTTCAGACGTGTGCTCTTCCGATC**GCCTAACGCTTGGCTTTCTC**

### Quality control of sequencing data and reads trimming

The quality of the data obtained after sequencing was assessed using the FastQC tool. When the score of the first bases of reads was lower than 30, all reads of the dataset were 5′-trimmed of the relevant number of nucleotides using the trimmomatic tool (Headcrop). All reads with more than 1 N-call were removed from datasets.

### ChIP-seq reads mapping, peak calling and analysis

ChIP-seq reads were aligned on the human reference genome (hg19) using CASAVA 1.8.2 (MGX pipeline). Analysis of the aligned reads, scaling and input subtraction were performed using the R package Pasha (35). Data were visualized using the IGB software (36). The peak calling was performed using the WigPeakCaller script, which automatizes the IGB thresholding tool (37). The SUMO-2/3 peak calling was done with the following parameters: by value = 32, Max Gap ≤100 and Min Run >100. Motif search was performed using HOMER v4.10 (38). ChIP-Seq sequencing data are available with accession GSE198986. Publicly available HL-60 ChIP-seq dataset were used for H3K4me3 (GSM945222), H3K4me1 (GSM2836484), H3K27ac (GSM2836486) and RNAPII (GSM1010737). The hg19 promoter (−2 kb to TSS) gff files have been generated with gff_toolbox, using the GRCh37p13 annotation file from NCBI. The H3K4me3 histone marks, which is enriched at gene TSS, have been used as a proxy to annotate HL-60 promoter. All genomic regions presenting H3K4me1, which do not correspond to annotated promoters, were considered as candidate enhancers. Then, the activity of these regulatory elements was inferred from the presence of H3K27ac. All dataset intersects were performed using Bedtools 2.29.0 (intersect) from Quinlan laboratory (39,40).

### RNA-seq mapping, quantification and differential analysis

RNA-seq reads were mapped to Human reference genome (hg19, GRCh37p13) using TopHat2 (2.1.1) (41) based on the Bowtie2 (2.3.5.1) aligner (42). The reproducibility of replicates was quantified using the cufflinks v2.2.1 tool (43) with the linear regression of reads per kilobase million (RPKM) between two replicates. Read association with annotated gene regions was done using the HTseq-count tool v0.11.1 (44). The variance between replicates and conditions were appreciated thanks to a principal component analysis (PCA) performed on the read count matrix. Differential expression analysis was performed using DESeq2 (45) using the normalization by the sequencing depth and the parametric negative binomial law to estimate data dispersion. All conditions were compared to the mock condition (DNR versus DMSO, ML-792 versus DMSO and ML-792 + DNR versus DMSO) and the ML-792 + DNR condition was also compared to the DNR-only condition (ML-792 + DNR versus DNR). The genes that presented a fold change ≥ or ≤ 2 and an adjusted *P*-value (FDR) <0.05 were considered as differentially expressed genes (DEGs). RNA-seq data are available with accession GSE198982.

### CUT&RUN mapping, trim and profiling

Reads were trimmed using Trimmomatic (v0.39) with paramaters ILLUMINACLIP:TruSeq3-SE.fa:2:30:10 LEADING:3 TRAILING:3 SLIDINGWINDOW:4:15 MINLEN:50. Reads were then aligned to GRCH38/hg38 human reference genome using Bowtie 2 (v2.3.5), converted to WIG and scaled with the PASHA pipeline (threshold 70 000, bin 50)(35). WIG files from the same replicates were merged using the mergeWigs R tool. Peaks were called using the PASHA pipeline (threshold 360, minRun 50, MaxGap 50). BigWig files were generated using the UCSC wigToBigWig tool (46). Heatmaps and metaprofiles were generated using deeptools (3.5.1) (47). When compared to CTCF signals, bigWig and bed files from SUMO2 and Histone marks were lifted over to hg38 using CrossMap (v0.6.5) (48). The CUTNRUN data are available with accession number GSE231023.

### 4C-seq mapping, trim, capture and profiling

The pipeline for the analysis of the 4C data was modified from the pipe4C pipeline (49) and is available on github (https://github.com/Mathias-Boulanger/pipe4C). The steps are the following: Reads filtering (trim-capture), mapping to reference genome, assignment of reads to their restriction fragment and creation of normalized score per fragment. Only reads containing the amplification sequence (CGTGACGCACGGAAACGTC) were kept for further analysis. Then, sequences downstream of the restriction enzyme cutting site of each selected reads were mapped to GRCh37p13 human reference genome with Bowtie2 aligner. Restriction fragment map was extrapolated from the reference genome using the cutting sequence of restriction enzymes. The interaction peak calling has been performed with peakC and the differential profiling analysis with DESeq2 (45,50). 4C-seq data are available with accession GSE198981.

### Gene ontology and GSEA

Functional gene-annotation enrichment analyses were done using GO Panther (51) with the ID number of DEGs or proteins as input list. The gene network analyses were performed using the Cytoscape-based Cluego plugin (52). Gene Set Enrichment Analyses were performed using https://www.gsea-msigdb.org/gsea/index.jsp (version 4.0.3) (53).

### Coupling antibodies to protein-G beads

Hybridoma supernatants were incubated with Protein G sepharose beads (SIGMA) at room temperature for 4 h, washed 3 times with PBS (phosphate buffer 10 mM pH 7.4, KCl 2.7 mM and NaCl 137 mM) and once with Na borate 50 mM pH 9.0. Antibodies were then crosslinked for 30 min in dimethyl-pimelimidate (DMP) 20 mM diluted extemporarily in Na borate 50 mM pH 9.0. The coupling procedure was repeated a second time and the beads were washed 3 times with PBS.

### Immunoprecipitation of SUMOylated proteins

For SILAC experiments, SILAC-labeled HL-60 cells were grown in spinner flasks (Nunc). 5 × 10^8^ cells were used for each condition. The immunoprecipitation of endogenously SUMOylated proteins was based on the protocol described in reference (54). Cells were lysed in PBS containing SDS 2%. The final concentration of SDS after lysis was then adjusted to 1% and lysates were sonicated. Dithiotreitol (DTT) was then added at a final concentration of 50 mM. Lysates were then boiled for 10 min and diluted 10-fold in Na phosphate 20 mM pH 7.4, 150 mM NaCl, Triton X100 1%, Na deoxycholate 0.5%, EGTA 5 mM, EDTA 5 mM, NEM, 10 mM, aprotinin + pepstatin + leupeptin 1 μg/ml each, filtered through 0.45 μm filter and incubated with Protein G-coupled anti-SUMO-1, -SUMO-2 and -BrdU (control) antibodies at 4°C overnight. Beads were then washed 3 times with RIPA (Na phosphate 20 mM pH 7.4, NaCl 150 mM Triton X100 1%, SDS 0.1%, Na deoxycholate 0.5%, EGTA 5 mM, EDTA 5 mM, NEM 10 mM, aprotinin1μg/ml and pepstatin 1 μg/ml) and twice with RIPA containing NaCl 350 mM in LowBind tubes (Eppendorf). Elution of SUMOylated proteins was performed twice with peptides bearing either the 21C7 SUMO-1- (VPMNSLRFLFE) or the 8A2 SUMO-2/3- (IRFRFDGQPI) epitope diluted in RIPA containing NaCl 350 mM. Eluted proteins were precipitated with 10% TCA for 1 h on ice. Pellets were then washed twice with acetone at -20°C, dried and resuspended in the Laemli electrophoresis sample buffer. For the identification of SUMOylated targets (SILAC1), samples were immunoprecipited with control-, anti-SUMO-1 or anti-SUMO-2/3 antibodies and mixed only after elution with the SUMO epitope-bearing peptides. For the identification of proteins showing DNR-modulated SUMOylation, mock- and DNR-treated samples were mixed right after the initial lysis step and used for immunoprecipitation with SUMO-1 (SILAC2) or SUMO-2/3 (SILAC3) antibodies.

### Mass spectrometry identification of SUMOylated proteins

Enriched SUMOylated proteins from SILAC lysates were size-separated by SDS-PAGE and in-gel digested with trypsin. The resulting peptide mixtures were extracted, desalted and concentrated on STAGE-tips with two C18 filters and eluted two times with 10 μl of acetonitrile 40% in formic acid 0.5% prior to online nanoflow liquid chromatography-tandem mass spectrometry (nano LC–MS/MS) using an EASY-nLC system (Proxeon, Odense, Denmark) connected to the Q Exactive HF (Thermo Fisher Scientific, Germany) through a nano-electrospray ion source. Peptides were separated in a 15 cm analytical column in-house packed with 1.9 μm C18 beads (Reprosil-AQ, Pur, Dr Manish, Ammerbuch-Entringen, Germany) using an 80 min gradient from 8% to 75% acetonitrile in acetic acid 0.5% at a flow rate of 250 nl/minute. The mass spectrometers were operated in data-dependent acquisition mode with a top 10 method. For Q-Exactive measurements, full scan MS spectra were acquired at a target value of 3 × 10^6^ and a resolution of 60 000 and the Higher-Collisional Dissociation (HCD) tandem mass spectra (MS/MS) were recorded at a target value of 1 × 10^5^ and with a resolution of 60 000 with a normalized collision energy of 30%.

Raw mass spectrometry (MS) files were processed with the MaxQuant software suite (version 1.4.0.3, www.maxquant.org). All resulting MS/MS spectra were searched against the human Uniprot database (www.uniprot.org) by the Andromeda search engine using the reversed database strategy applying a false discovery rate of 0.01 at both peptide and protein levels. Overrepresentation of Gene Ontologies of the identified proteins were analyzed using Fisher's exact test from InnateDB (55).

### Statistical analyses

Results are expressed as means ± S.D. Statistical analyses were performed using Anova or paired Student's *t*-test with the Prism 9 software. Differences were considered as significant for *P*-values of <0.05. *, **, ***, **** correspond to *P* < 0.05, *P* < 0.01, *P* < 0.001, *P* < 0.0001, respectively. ns = not significant. Statistical analyses of the transcriptomic and proteomic experiments are described in the relevant sections.

## RESULTS

### DNR rapidly induces transcriptional programs related to cell proliferation/death and inflammation/immunity in AML cells

**Figure 1.**
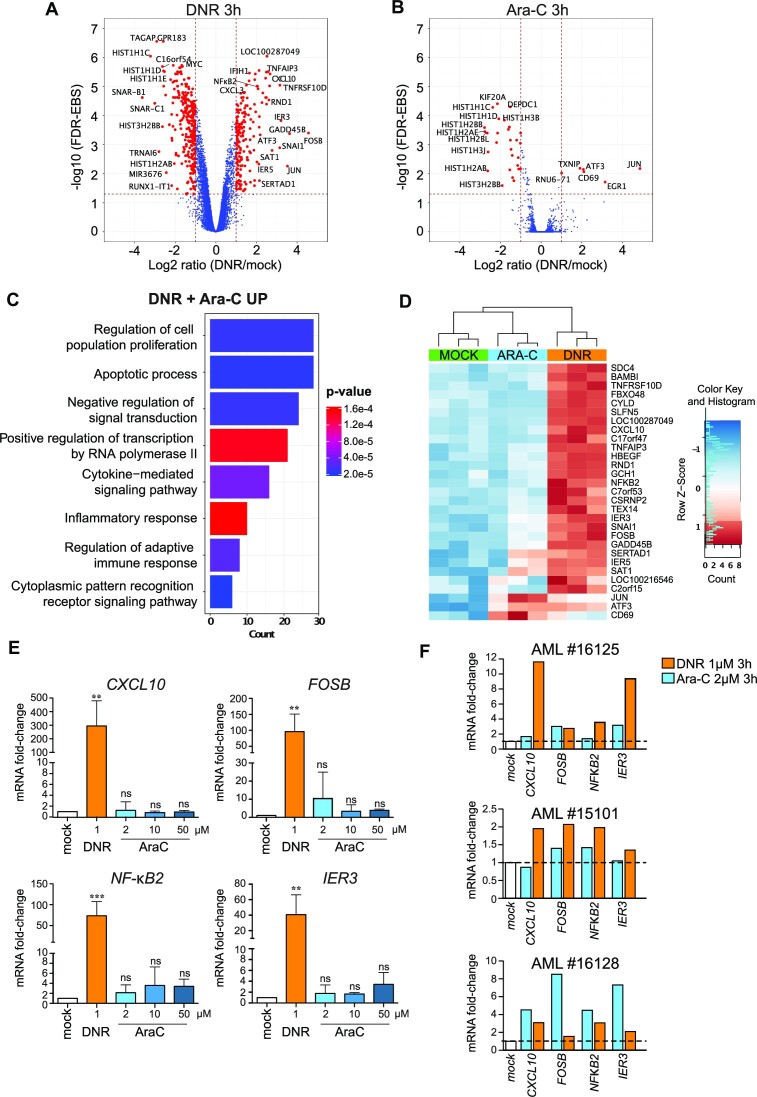
Chemotherapeutic drugs rapidly alter the expression of genes involved in cell death and inflammation in AML cells. (A, B) *Trancriptome profile*. HL-60 cells were treated with 1 μM DNR (**A**) or 2 μM Ara-C for 3 h (**B**). RNAs were purified from three independent experiments and used to probe Affymetrix Human Gene 2.0 ST Genechips. The red dots on the Volcano plots represent the Differentially Expressed genes (DEG) with an absolute Fold Change (FC) ≥ 2 (log_2_ ≥ 1) and a False Discovery Rate (FDR) corrected with Empirical Bayes Statistics (EBS) (89) <0.05. (**C**) *Gene Ontology enrichment analysis of the genes up-regulated (*≥*2 fold) by DNR and Ara-C*. Ontologies were performed using the Panther GO database (51). The main terms of each identified group are presented on the graph and classified by the number of genes present in each group. *P* values are corrected with Bonferroni step down. (**D**) *Heatmap of DEG with a FC*≥*4 in the transcriptomic experiments presented in (A) and (B)*. The data for all three replicates are represented. (**E**) *RT-qPCR analysis of selected genes*. HL-60 cells were treated for 3 h with 1 μM DNR or 2 μM Ara-C. The levels of the indicated mRNAs were measured by RT-qPCR, normalized to *GAPDH* levels and expressed as fold increase to mock-treated cells (mean ± SD, n = 7 for *NF-κB2*, *n* = 6 for *IER3*, *n* = 5 for *FOSB*, *CXCL10*). (**F**) *Regulation of selected genes in primary AML cells*. AML cells (bone marrow aspirate) from three patients were treated *in vitro* with 1 μM DNR or 2 μM Ara-C for 3 h. The levels of the indicated mRNAs were measured by RT-qPCR, normalized to *TBP* levels and expressed as fold increase to mock-treated cells.

To identify the genes whose expression is rapidly altered by Ara-C or DNR in AML cells, we performed a whole transcriptome profiling of HL-60 cells, one of the most widely used cellular model of AML (56). Cells were treated with each one of the two drugs at doses relevant to the clinical practice (2 and 1 μM, respectively) (57,58) for 3 h, i.e. before the onset of apoptosis, which begins after 4 h of treatment (13). Using the Affimetrix array technology, we identified 476 significant differentially expressed genes (DEGs) in DNR-treated cells, 182 being upregulated and 294 downregulated >2-fold (Figure 1A and Supplementary Table 1). Much less DEGs were identified in Ara-C-treated cells: 6 were upregulated and 29 downregulated by a >2-fold factor (Figure 1B). Gene ontology (GO) enrichment analyses revealed that the genes identified as down-regulated upon treatment by Ara-C and/or DNR are mostly involved in nucleosome assembly (Supplementary Figure 1A). Those up-regulated principally belong to functional categories linked to signal transduction, transcription, cell proliferation and death (with both pro- and anti-apoptotic genes being induced) and inflammation/immunity (Figure 1C, D, Supplementary Figure 1B, Supplementary Table 1). We confirmed the activation of four of the most DNR-induced genes (*CXCL10*, *FOSB*, *NFKB2* and *IER3*) by RT-qPCR in HL-60 cells treated with DNR (Figure 1E). Noteworthy, these genes were not significantly induced by Ara-C even at concentrations higher than 2 μM (up to 50 μM) (Figure 1E). Taken with our Affymetrix data (Figure 1A and B), this suggested that DNR is more potent at altering transcription than Ara-C in the HL-60 cell model. We then analyzed samples from three AML patients taken at diagnosis (Supplementary Table 2). These were treated *ex vivo* with DNR or Ara-C for 3 h and assayed for the expression of the same four genes. All of them were induced by DNR in the three patients tested, albeit to different degrees. Their expression was more induced by DNR than by Ara-C for two patients, showing that our observation in HL60 cells reflected a situation happening in primary AML cells. However, the reverse was observed for the third patient sample, which is probably reflecting AML heterogeneity (Figure 1F). Finally, we analyzed the effect of DNR and Ara-C on the expression of the same genes in Peripheral Blood Mononucleated cells (PBMC) from three different healthy donors. Only NFKB2 was induced in all three donors, at however lower levels than in AML patients’ cells (Supplementary Figure 1C).

Thus, our data indicate that one early effect of the chemotherapeutics used as frontline treatment of AML is transcriptional reprogramming. DNR, however, shows much broader effects than Ara-C and the genes most induced by DNR principally belong to two general functional categories: cell proliferation/death and inflammation/immunity.

### DNR induces a fast removal of SUMO from chromatin, in particular at active promoters and enhancers

**Figure 2.**
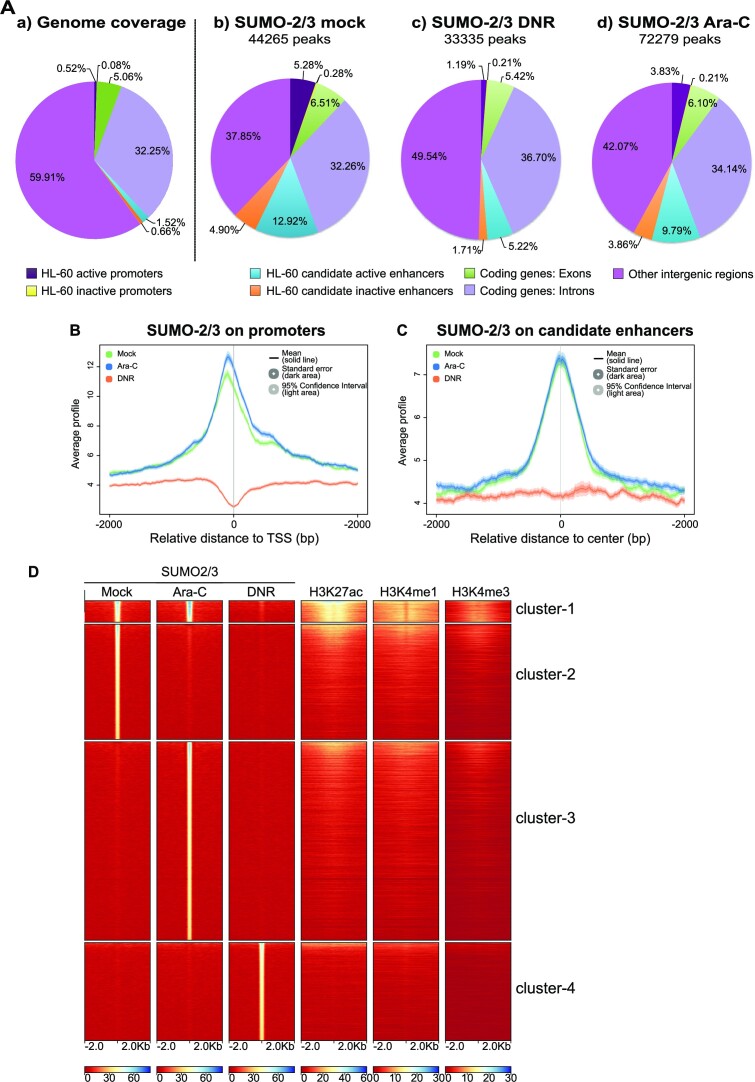
Treatment of AML cells with DNR depletes SUMOylated proteins from the chromatin, in particular at promoters and enhancers. (A, B) *ChIP-Seq analyses of SUMO-2/3 distribution on the genome*. HL-60 were treated with 1 μM DNR or 2 μM Ara-C for 2 h. ChIP-Seq experiments were carried out with SUMO-2/3 antibodies. (**A**) a: a proportion of the different genomic regions, b−d: proportion of SUMO-2/3 peaks on these chromatin regions in mock- (b), DNR- (c) or Ara-C- (d) treated HL-60 cells. (**B**, **C**) *Metaprofile of the SUMO-2/3 ChIP-seq signal* on HL-60 promoters (B) or enhancers (C) in mock-, DNR- or Ara-C- treated HL-60 cells. Promoters (−2 kb to TSS) and enhancers as well as their activation state were defined using H3K27ac, H3K4me1 and H3K4me1 profiles as well as NCBI refseq data (see Material and methods and Supplementary Figure 2). (**D**) Heat-map for the distribution of SUMO-2/3, H3K27ac, H3K4me1, H3K4me3. The clustering was performed on SUMO peaks present in any of the conditions (Mock, DNR, Ara-C) and the ranking was made according to SUMO-2/3 signal.

We have previously shown that DNR and Ara-C induce a progressive *de*SUMOylation of proteins in AML. It is due to the inactivation of the SUMO E1 and E2 enzymes via the formation of a ROS-dependent disulfide bond between their catalytic cysteines (13). Due to the role of SUMOylation in transcription, we wondered whether DNR and Ara-C could induce fast alterations in SUMOylated protein distribution on the genome, as such changes might regulate, positively or negatively, drug-induced transcriptional changes. This was addressed in ChIP-Seq experiments with antibodies directed to SUMO-2/3. HL-60 cells were treated with DNR or Ara-C for 2 h, *i.e*. a time point earlier than that used in our transcriptomic analysis to consider the time required between gene transcription alteration and RNA accumulation changes in the cell. In untreated cells, and as previously shown by others (17,18,21,25,59,60), SUMO-2/3 was found distributed all along chromatin with approximately 44 000 peaks (Figure 2A). A particular enrichment was found at both annotated gene promoters and candidate enhancer regions defined by the presence of high H3K27ac, H3K4me1 and low H3K4me3 (Supplementary Figure 2). In mock-treated cells, we identified 6861 genes showing a significant accumulation of SUMOylated proteins in their promoter regions with a peak of enrichment approximately 100 bp upstream of Transcription Start Sites (TSSs). Interestingly, SUMOylated proteins were found enriched on active promoter regions (those with high H3K4me3 and RNApolII) and not on inactive ones (those with low H3K4me3 and RNAPolII) (Supplementary Figure 2A). Along the same line, SUMOylated proteins were found localized in the center of the candidate enhancer regions and slightly more enriched on active- (i.e. with high H3K27ac) than on inactive- (i.e. with low H3K27ac) candidate enhancers (Supplementary Figure 2B).

We then analyzed whether DNR and Ara-C treatments globally affected the presence and/or the distribution of SUMO-2/3-conjugated proteins on chromatin. At promoters (Figure 2B) and enhancers (Figure 2C), levels of SUMOylation remained essentially unchanged upon Ara-C treatment (Figure 2D, cluster 1). In the other regions of chromatin, most SUMO-2/3 peaks disappeared upon Ara-C treatment (Figure 2D, cluster 2) and were redistributed to other genomic regions, resulting in an increase in the total number of SUMO peaks (Figure 2D, cluster 3). However, the global distribution of SUMO-2/3 peaks between chromatin regions remained similar upon Ara-C treatment (Figure 2Ad) and the average SUMO-2/3 peaks intensity remained unchanged (Supplementary Figure 3A). By contrast, DNR treatment induced a 25% decrease in the total number of SUMO-2/3 peaks (Figure 2Ac) as well as a decrease in the average SUMO-2/3 peak intensity (Supplementary Figure 3A). Most chromatin regions lost SUMOylation (Figure 2D) but the decrease was particularly strong at promoters (Figure 2B) and enhancers (Figure 2C). Similar to Ara-C treatment, new SUMO-2/3 peaks appeared upon DNR treatment in regions devoid of active transcription marks (Figure 2D, cluster 4). As mentioned earlier, the bulk of protein SUMOylation is not detectably affected at 2 h of DNR treatment (Supplementary Figure 4A). This raises the idea that chromatin-bound proteins, in particular those enriched at gene *cis*-regulatory regions are among the first proteins to be *de*SUMOylated upon DNR treatment. At this early time point, DNR has already induced some DNA damage as measured by γH2AX staining (Supplementary Figure 4B). However, Annexin-V labelling shows that cells have not yet entered into apoptosis, which starts after 4 h of treatment (Supplementary Figure 4C).

### Inhibition of SUMOylation limits both positive and negative changes in gene expression induced by DNR

**Figure 3.**
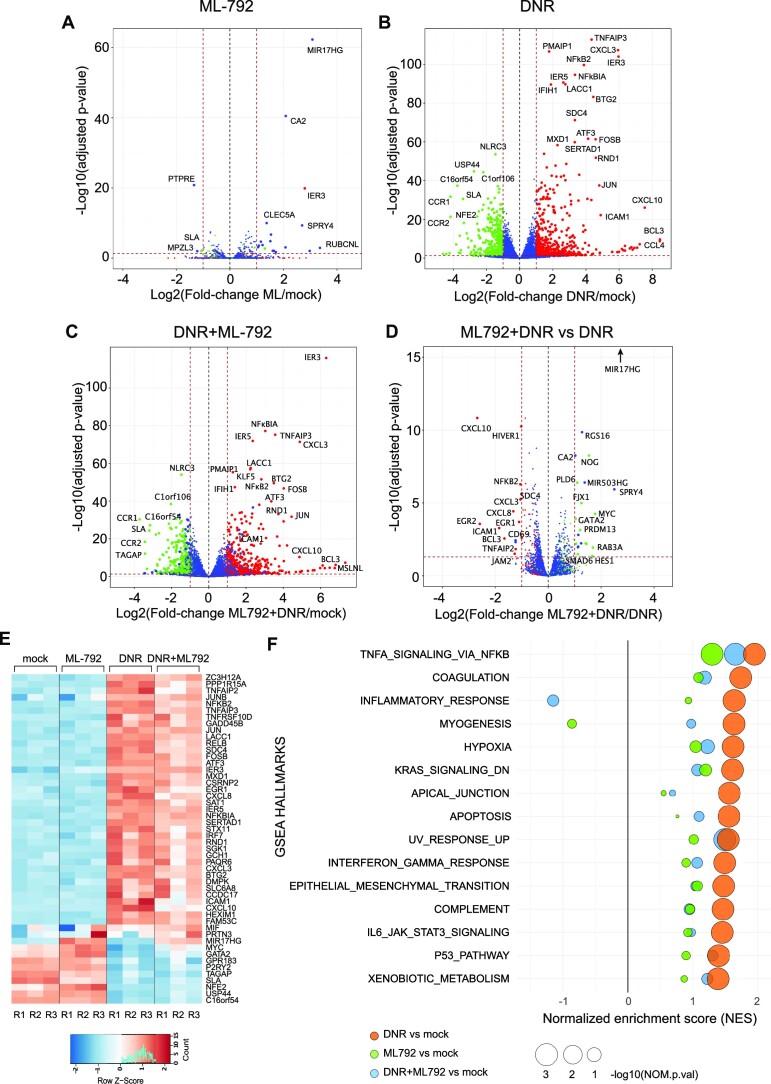
Inhibition of SUMOylation reduces the DNR-induced regulation of a subset of genes. (A–E) HL-60 cells were treated with 1 μM DNR, 0.5 μM ML-792 or the combination of the two drugs for 3 h. Total RNAs were prepared from three independent experiments and sequenced. Volcano plot showing the DEG between (**A**) ML-792- and mock- (**B**) DNR- and mock-, (**C**) ML-792 + DNR- and mock-, (**D**) ML-792 + DNR and DNR- treated HL-60 cells. Green dot: DNR-downregulated FC ≤ -2 and FDR (false discovery rate) < 0.05; red dots: DNR-upregulated with FC ≥ 2 and FDR < 0.05; Blue dots: genes with –2 ≥FC ≤ 2 and FDR > 0.05 in the DNR *vs* mock conditions. (**E**) Heatmap of top 50 DEGs in all conditions presented in A, B and C. (**F**) Gene Set Enrichment Analysis (GSEA) was performed using RNA-Seq data presented in A–E. The GSEA hallmarks showing a Normalized Enrichment Score NES > 1 or < −1, a *P*-value < 0.05 and an FDR < 0.25 for the DNR versus mock analysis are presented for each treatment condition (DNR, ML-792, DNR + ML-792) compared to the mock-treated cells.

As DNR had much stronger effects on chromatin SUMOylation and gene expression than Ara-C, we continued our investigations by assessing whether inhibition of SUMOylation is sufficient to induce the expression of DNR-responsive genes. To this aim, we performed RNA-seq analyses of HL-60 cells treated for 3 h with the highly potent and selective SUMOylation inhibitor ML-792 (61). Upon ML-792 treatment, all SUMO-2/3 targets were deconjugated after one hour (Supplementary Figure 4D). Surprisingly, ML-792 had minimal effect on gene expression with only 21 differentially regulated genes (Figure 3A), suggesting that *de*SUMOylation *per se* is not sufficient to induce DNR-responsive genes. As there is no specific *de*SUMOylation inhibitors that could be used to prevent DNR-induced *de*SUMOylation, we used ML-792 in combination with DNR to strengthen and accelerate DNR-induced *de*SUMOylation. RNA-Seq being more sensitive than the Affimetrix array-based approach, we identified more DNR-responsive genes than in our former transcriptomic approach (Supplementary Figure 4E). 552 genes were found up-regulated and 380 down regulated in DNR *vs* mock-treated cells (Figure 3A and Supplementary Table 3). The level of up- or down-regulation was not correlated to the level of change in SUMO-2/3 levels present at their promoters upon DNR treatment (Supplementary Figure 3B). Nevertheless, the comparison of ML-792 + DNR- to DNR only-treated-cells revealed that inhibition of SUMOylation during the DNR treatment generally limited DNR-target genes up- or down-regulation (Figure 3C, D). This was in particular the case for the genes, which are the most affected by DNR (Figure 3D and E). GSEA analysis showed that all pathways enriched in DNR-treated cells were less or not enriched at all when SUMOylation was inhibited, the most pronounced effects being observed for the genes involved in inflammation (Figure 3F and Supplementary Table 4). Thus, our data suggest that inhibition of SUMOylation counteracts the ability of DNR to alter the expression of its responsive genes, whether induced or down-regulated.

### Transcription factors and co-regulators are the fastest and main class of *de*SUMOylated proteins upon DNR treatment

**Figure 4.**
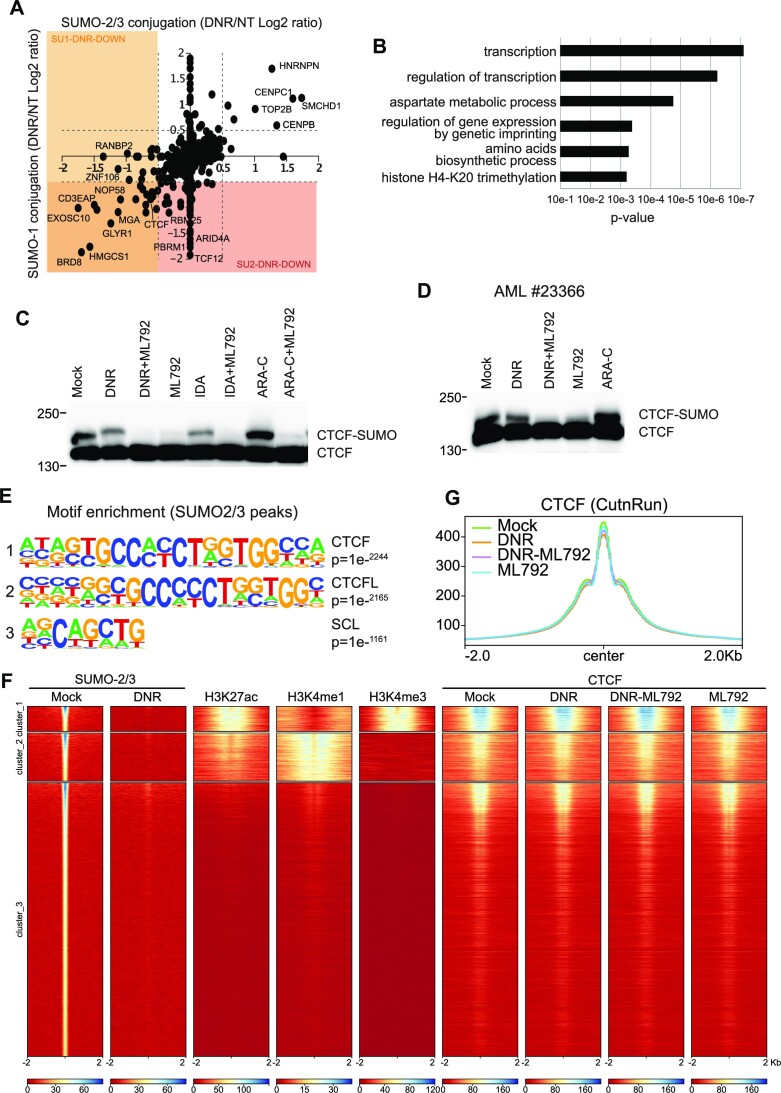
DNR leads to *de*SUMOylation of chromatin regulators, including CTCF. (**A**) *Changes in SUMO-1 and SUMO-2/3 proteomes upon DNR treatment*. SUMOylated proteins were immunoprecipitated with SUMO-1 or SUMO-2/3 antibodies from SILAC-labeled HL-60 cells treated or not with DNR (1 μM for 2 h). Scatterplot analysis of SUMO-1 and SUMO-2/3 proteome change (log_2_ ratio) in cell treated compared to mock-treated cells. Doted lines represent log_2_ ratio of ±0.5. Only proteins found to be SUMOylated (Supplementary Figure 4A) are represented. (**B**) *DeSUMOylated proteins are mostly transcriptional regulators*. Gene Ontology analysis of the identified down-SUMOylated proteins for SUMO-1 and SUMO-2/3 in response to DNR (log_2_ ratio < −0.5) were obtained using the Panther Protein Class database (51). (C, D) *CTCF is SUMOylated in HL-60 and patient cells*. HL-60 (**C**) or AML patient cells (**D**) were treated with DNR (1 μM), ML-792 (0.5 μM), IDA (1 μM) or Ara-C (2 μM) for 3 h. Total cell extracts were loaded on SDS-PAGE and immunoblotted with CTCF antibodies. (**E**) *The CTCF motif is enriched at SUMO-2/3 binding sites*. Motif enrichment search was performed with homer pearl script (findMotifs.pl) on the SUMO-2/3 ChIP-Seq data obtained for mock-treated HL-60. The three most enriched motifs are shown. (**F**) *SUMO/CTCF overlap on promoters and enhancers*. HL-60 cells were treated with DNR (1 μM), ML-792 (0.5 μM) or the combination for 2 h. Cell extracts were then used to perform CUT&RUN with CTCF antibodies (three independent biological replicates). Heat-map for the distribution of SUMO-2/3 (ChIP-Seq, see Figure 2), H3K27ac, H3K4me1, H3K4me3 and CTCF (CUT&RUN). The clustering was performed on H3K4me1 and H3K4me3 and the ranking was made according to SUMO-2/3 signal. (**G**) *Metaprofile for the distribution of CTCF* peaks on the whole genome in cells treated for 2 h with mock, DNR (1 μM), ML-792 (0.5 μM) or the combination.

To better understand how *de*SUMOylation controls DNR-responsive gene expression, we next resorted to large-scale proteomics to identify the proteins changing their SUMOylation levels after 2 h of DNR treatment, i.e. the time point at which important changes in chromatin protein SUMOylation were detected by ChIP-seq (Figure 2). First, we characterized the HL-60 cell proteome conjugated to SUMO-2/3 but also to SUMO-1. To this aim, we immunoprecipitated and identified by quantitative mass spectrometry SUMO-2/3 and SUMO-1 modified proteins. 894 SUMO targets were identified, most of them being modified by both SUMO-2/3 and SUMO-1 (Supplementary Figure 5A). Then, SUMO-2/3 or SUMO-1-conjugated proteins were immunoprecipitated and identified from HL-60 cells treated or not with DNR for 2 h. As expected from immunoblotting experiments (Supplementary Figure 4A), the SUMOylation level of most proteins did not change after 2 h treatment with DNR. However, 34 proteins (31 for SUMO-2/3 and 11 for SUMO-1, 8 proteins being common) showed increased modification (Figure 4A and Supplementary Table 5). More proteins (83 for SUMO-2/3 and 32 for SUMO-1, 19 being common) showed a significant decrease in their SUMO conjugation upon DNR treatment (Figure 4A and Supplementary Table 5). Finally, these changes were not due to modifications of protein abundance, as determined by sequencing of input samples in control- and DNR-treated cells (Supplementary Figure 5B and Supplementary Table 5). Interestingly, after 2 h of treatment, most of the *de*SUMOylated proteins (both SUMO-2/3 and SUMO-1 substrates) were found to be chromatin-bound proteins involved in the regulation of gene expression (Figure 4B).

Thus, our proteomic data support the idea initially raised by our SUMO-2/3 ChIP-seq experiments (Figure 2) that chromatin-bound proteins are among the first to be *de*SUMOylated upon treatment by DNR.

### CTCF colocalizes with SUMO on chromatin, in particular on active *cis-*regulatory regions, and is *de*SUMOylated upon DNR treatment

Among the SUMOylated substrates found *de*SUMOylated upon DNR treatment in the SILAC experiment (Figure 4A), we noted the CCCTC-binding factor CTCF, an insulator protein known to regulate the three-dimensional architecture of chromatin (62). CTCF was formerly reported to be SUMOylatable (63) and its SUMOylation to be instrumental for activation and repression of the *PAX6* (64) and *c-MYC* (65) genes, respectively. We first confirmed the SUMOylation of CTCF by the presence of a band migrating above CTCF on SDS-PAGE, which disappeared upon SUMOylation inhibition with ML-792 in both HL-60 (Figure 4C) and primary AML patient's cells (Figure 4D). DNR as well as the other anthracycline Idarubicin (IDA) induced a decrease in CTCF SUMOylation, whilst Ara-C had no effect (Figure 4C and D). In addition, we found that the most represented DNA-binding motif under the SUMO peaks identified in our ChIP-seq experiments (Figure 2) was the consensus CTCF-binding motif (Figure 4E and Supplementary Table 6). To further confirm the link between SUMO and CTCF, we performed CUT&RUN experiments with CTCF antibodies to map CTCF binding sites in HL-60 cells. This showed a strong colocalization between SUMO and CTCF binding on the chromatin, with around one third of SUMO-bound regions being bound by CTCF (Figure 4F). The strongest co-localization was found at chromatin regions presenting marks of active transcription (Figure 4F). This is in particular the case around gene TSSs, which are losing SUMOylation upon DNR but not Ara-C treatment (Supplementary Figure 6A). Treatment with DNR, ML-792 or their combination did not significantly affect CTCF distribution on the chromatin (Figure 4G) suggesting that decreased SUMOylation of CTCF and other chromatin-bound protein does not induce the offloading of CTCF from chromatin.

### SUMOylation regulates DNR-induced expression of the CTCF and SUMO-bound *NFKB2* gene

**Figure 5.**
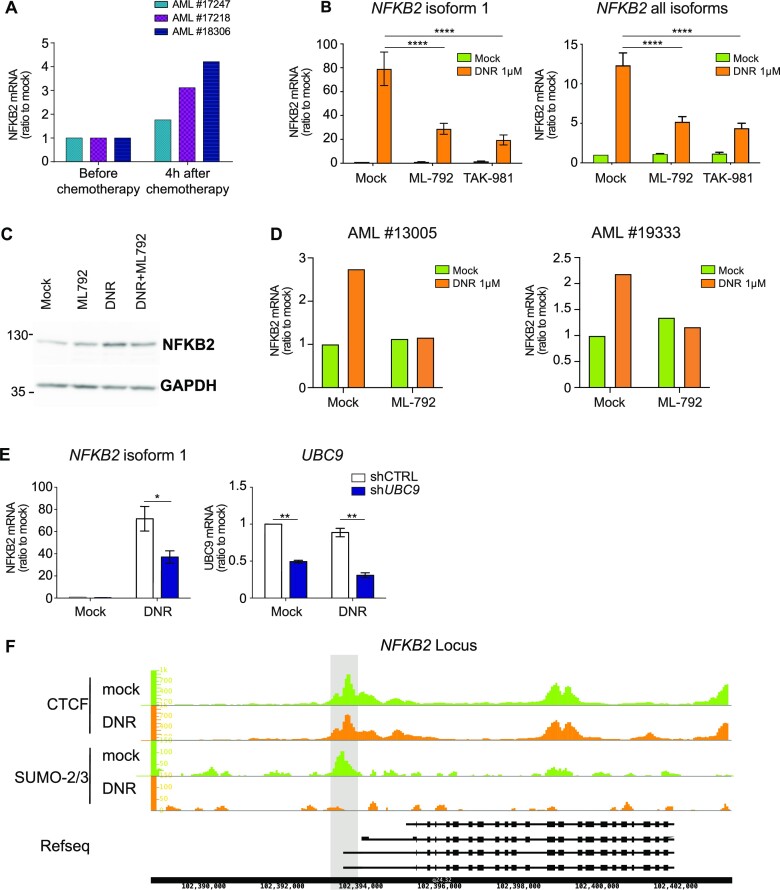
*de*SUMOylation limits DNR-induced changes in *NFKB2* expression. (**A**) *Regulation of NFκB2 gene during AML patient treatment*. Blood sample from three patients were collected before and 4 h after the induction chemotherapy (DNR: 90 mg/m^2^ and Ara-C 30 mg/m^2^). PBMC were purified, mRNA prepared and *NFKB2* expression monitored by RT-qPCR, normalized to TBP and S26 levels and expressed as ratio to cells before treatment. (**B**) *inhibition of SUMOylation limits NFκB2 induction by DNR*. HL-60 cells were treated with 1 μM of DNR for 3 h with or without 0.5 μM of ML-792 or 0.5 μM TAK-981. The levels of the indicated mRNAs were measured by RT-qPCR, normalized to TBP and S26 and expressed as ratio to mock-treated cells (*n* = 6 for DMSO and ML-792, *n* = 3 for TAK-981, Ordinary One-Way Anova). (**C**) *inhibition of SUMOylation limits NFκB2 protein accumulation upon DNR treatment*: HL-60 cells were treated with 1 μM of DNR for 3 h with or without 0.5 μM of ML-792. Cell extracts were loaded on SDS-PAGE and immunoblotted with NF*κ*B2 and GADPH antibodies (*n* = 3). (**D**) *Inhibition of SUMOylation limits NFκB2 induction by DNR in AML patient cells*. AML cells (bone marrow aspirates) from two different patients were treated with 1 μM of DNR for 3 h with or without 0.5 μM of ML-792. The levels of *NFKB2* mRNAs were measured by RT-qPCR, normalized to *GAPDH* and expressed as ratio to mock-treated cells. (**E**) *UBC9 knock-down limits DNR-induced NFKB2 expression*. HL-60 cells stably expressing scramble or *UBC9* directed shRNA were mock- or DNR-treated for 3 h. The levels of the indicated mRNAs were measured by RT-qPCR, normalized to TBP and S26 and expressed as ratio to mock-treated cells (*n* = 3). (**F**) *CTCF and SUMO bind to the NFκB2 promoter:* ChIP-Seq data for SUMO-2/3 and CUT&RUN data for CTCF were aligned and visualized using the IGB software at the level of the *NFKB2* gene.

To further investigate the link between CTCF and SUMO in DNR-induced gene expression changes, we crossed the list of genes presenting SUMOylated proteins and CTCF in their promoters with that of genes transcriptionally affected more the 2-fold upon DNR treatment. Sixty-one genes were identified, the expression of which might be regulated through SUMOylation/*de*SUMOylation of proteins bound to their promoter regions (Supplementary Figure 6B, left panel). We then crossed this list with that of the 36 genes whose DNR-induced expression changes was altered by more than 2-fold upon SUMOylation inhibition (Supplementary Figure 5B, right panel). This led to the identification of four genes (*EGR1*, *ICAM1*, *MYC* and *NFKB2*) whose DNR-induced up- or down-regulation is reduced upon inhibition of SUMOylation and whose proximal promoters are marked by SUMO and CTCF. We then focused on the *NFKB2* gene, encoding the transcription factor Nuclear Factor-kappa B2 (NF-κB2), because of its involvement in the regulation of both cell death/survival and inflammation/immunity (66,67), processes we found associated with the response of AML to DNR. Moreover, after having formerly shown that DNR induces *NFKB2* expression in AML patients’ cells treated *in vitro* (Figure 1F), we established the early induction of this gene *in vivo* using peripheral blood mononuclear cells (PBMCs) purified from 3 AML patients before and 4 h after the beginning of an induction chemotherapy comprising DNR and Ara-C (Figure 5A). Using HL-60 cells, we could show that the DNR + Ara-C combination was however not more efficient then DNR alone at inducing *NFKB2*. The other anthracycline IDA was also inducing *NFKB2*, at even higher levels than DNR (Supplementary Figure 6C). Finally, higher induction levels were detected when considering only *NFKB2* longest isoform, which starts at the CTCF/SUMO bound site (Figure 5B). Consistent with our RNA-Seq data (Figure 3), the SUMOylation inhibitor ML-792 decreased the DNR-induced expression of *NFKB2*. Similar results were obtained with another SUMOylation inhibitor, TAK-981 (68) (Figure 5B). In addition, DNR led to the accumulation of NFKB2 protein, which was limited by ML-792 (Figure 5C). Importantly, ML-792 also prevented the induction of *NFKB2* by DNR in primary AML cells from 2 patients treated *ex vivo* (Figure 5D). To further confirm the implication of SUMOylation inhibition in this process, we resorted to RNAi to down-regulate the SUMO E2 enzyme Ubc9. This did not affect the basal level of *NFKB2* expression but limited its DNR-induced up-regulation (Figure 5E). ChIP-Seq data identified a major SUMO-2/3 peak colocalizing with CTCF at the most 5′ promoter of *NFKB2* in HL-60 cells (Figure 5F), which disappeared upon DNR treatment. However, consistent with the genome wide results, DNR did not affect the binding of CTCF to the *NFKB2* gene (Figure 5F). Thus, altogether, our results suggest that *de*SUMOylation limits DNR-induced expression of the CTCF-bound *NFKB2* gene without modifying CTCF binding to the locus in AML cells.

### *De*SUMOylation limits DNR-induced chromatin 3D rearrangements at the *NFKB2* locus

**Figure 6.**
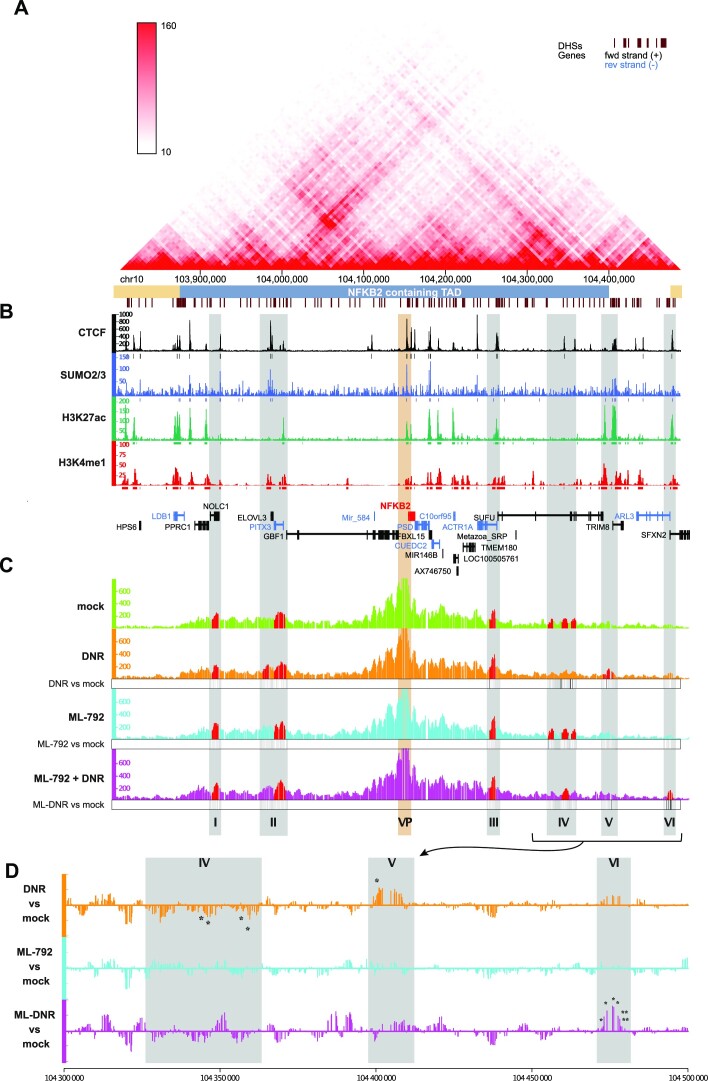
*de*SUMOylation limits DNR-induced changes in the 3D conformation of the *NFκB2* locus. (**A**) *HiC map of the TAD containing NFκB2 gene*. This map was obtained using publicly available HiC data obtained in the K562 human chronic myeloid cell line (90). The *NFKB2*-containing TAD is underlined in blue. (**B**) *Distribution of SUMO and CTCF in the NFκB2 containing TAD*. CUT&RUN data for CTCF and ChIP-Seq data for SUMO-2/3, H3K27ac and H3K4me1 are represented by the normalized read count per 50 bp bin (**C**, **D**) *Inhibition of SUMOylation limits DNR-induced changes in NF-κB2 locus 3D conformation*. HL-60 treated for 2 h with DNR (1 μM), ML-792 (0.5 μM) or the combination and subjected to 4C experiment (three biological replicates). The Y axis of the 4C-seq tracks represents the normalized interaction frequencies with the viewpoint (*NFKB2* promoter, VP) per 10 bp bin. Grey zones are highly reproducible interaction region in at least one condition (regions plotted in red present a *P*-value <0.05 in the peakC analysis of the three replicates) and named from I to VI. (D) Differential analysis of the contact point frequency in the regions IV-VI for DNR, ML-792 and ML-792 + DNR compared to mock-treated cells. *p*-values for the peaks showing statistically significant differences between the conditions are indicated.

Publicly available HiC data indicate that *NFKB2* is located at the center of a Topologically-Associating Domain (TAD), which extends over 500 kb on chromosome 10 (Figure 6A). They also suggest the existence of various long-range interactions between the *NFKB2* gene and distant regions within this TAD. Moreover, CTCF largely co-localizes with SUMO-2/3 in HL-60 cells, not just at the *NFKB2* locus, but also at various places covering the whole *NFKB2* TAD (Figure 6B). Together, these observations suggested that DNR-induced *NFKB2* expression could be associated with changes in chromatin organization that could be regulated by SUMOylation/*de*SUMOylation events.

To address this point, we resorted to Circularized Chromatin Conformation Capture (4C) experiments in HL-60 cells, using the *NFKB2* promoter as a viewpoint. In mock treated cells, we found that this promoter interacts significatively with two regions upstream of the *NFKB2* gene (regions I and II) and two downstream of it (regions III and IV) (red domains in the upper lane of Figure 6C). Noteworthy, they were all localized within the *NFKB2* TAD in the hundred kb-range from the *NFKB2* TSS and presented at least one CTCF-bound site.

The overall topology of the *NFKB2* locus was not strongly affected by a 2 h treatment with DNR (compare green and orange profiles in the first two lanes of Figure 6C). However, a differential profiling analysis (Figure 6D) showed decreased interactions between the CTCF/SUMO-bound *NFKB2* promoter and region IV in DNR-treated cells. Moreover, DNR induced a new interaction with the region V localized at the extreme border of the *NFKB2* TAD (Figure 6C and D). Interestingly, this new interacting region is enriched for histone marks characteristic of active enhancers (H3K27ac and H3K4me1), while the interacting region IV in mock-treated cells was devoid of such marks (Figure 6B). Thus, DNR-induced up-regulation of *NFKB2* is associated with changes in the frequencies of chromatin looping between its promoter region and distal regions within the *NFKB2* TAD, which include a potential enhancer.

To assess whether the SUMO pathway could be involved in chromatin 3D organization changes induced by DNR at *NFKB2* locus, we also conducted 4C experiments on cells treated with ML-792 alone or in combination with DNR. Treatment with ML-792 alone, which did not affect *NFKB2* gene expression, did not modify the overall 4C profile of the locus (compare green and blue profiles in lanes 1 and 3 of Figure 6C and see differential profiling in Figure 6D). However, when used together with DNR, ML-792 prevented the changes observed in the presence of DNR only (*i.e*. reduction of interactions with regions IV and induction of interaction with region V) and led to a new interaction with region VI surprisingly localized beyond the *NFKB2* TAD border (Figure 6C and D). Taken together, our data suggest that *de*SUMOylation of proteins bound at CTCF-bound sites in the *NFKB2* promoter limits *NFKB2* activation by DNR by affecting the chromatin 3D architecture changes induced by DNR at this locus.

## DISCUSSION

In this work, we report that an early effect of DNR, one of the two frontline chemotherapeutics used in AML treatment, is an alteration of specific transcriptional programs. DNR modifies the expression of almost 1000 genes in chemosensitive HL-60 cells after only 3 h of treatment. In contrast, much less genes are regulated by Ara-C. Importantly, selected DNR-up-regulated genes were also rapidly induced in three primary AML patient samples and one of them (*NFKB2*) was also rapidly upregulated *in vivo* during standard AML chemotherapy. However, besides this, the top DNR-up-regulated genes found in HL-60 cells were more induced by Ara-C than by DNR in one of the AML primary samples whereas they were hardly induced by Ara-C in the two other samples. Thus, altogether, our data indicate that DNR and Ara-C induce rapid (hour-range) transcriptome changes in AML with the effect of DNR being much stronger than those of Ara-C. However, at the same time, they also suggest a certain degree of variability between AML patients that is likely explained by AML heterogeneity.

One of the main pathways we found associated with DNR-up-regulated genes is apoptosis. This suggests that the rapid gene expression changes induced by this drug set up a favorable pro-apoptotic ground that adds to the DNA damages it generates for killing chemotherapy-treated AML cells at a later stage. It should, however, be noted that, in addition to pro-apoptotic genes, anti-apoptotic ones were also activated. This observation is consistent with those by others that DNR also activates pro-survival PI3-K/AKT- and NF-κB pathways and that their targeting is considered as a potential therapeutic strategy to improve their efficiency (6,66). Another functional category found enriched in DNR-induced genes was inflammation and immunity-related processes. In various immunocompetent mouse models, antracyclines were described as capable of inducing the immunogenic cell death of diverse solid tumors, in particular through the induction of an interferon response (69–71). The genes we identified as up-regulated in DNR-treated AML cells could participate in the development of an adaptative immune response against leukemic cells in chemotherapy-treated patients. Finally, downregulated genes are highly enriched for histone genes. This could result in decreased histone levels, which might loosen chromatin and favor the genotoxic action of the chemotherapeutic drugs. Altogether, our data suggest that the fast transcriptome changes induced by DNR before treated cells start dying may contribute to the response of AML to this drug. The molecular mechanisms underlying the effect of anthracyclines on gene expression are however far from being understood and probably multiple. Anthracyclines induce histone eviction at open chromatin regions, which were proposed to participate to the regulation of gene expression (72). DNA-damage induced by anthracyclines could also modulate gene expression. However, DNA-damages, in particular double strand breaks, are known to stall RNA-polymerase II at the break point and cause a global transcriptional shut down (73). It is therefore unlikely that DNR-induced transcriptional reprogramming, in particular gene up-regulation, is directly due to DNA damage. Nevertheless, DNA-damage-induced activation of specific transcription factors could participate to the activation of specific genes. Finally, anthracyclines are known to generate ROS, which functions as second messengers via the reversible oxidation of catalytic cysteines to activate many signaling pathways (74). Although anthracyclines-induced ROS generation has mostly been studied in cardiomyocytes due to their key role in anthracyclines cardiotoxicity (75), it is expected that they activate signaling pathways in cancer cells. Among the targets of anthracyclines-generated ROS are the SUMO E1 and E2 enzymes, whose respective catalytic cysteines form a reversible disulfide bridge upon oxidation, inhibiting their ability to activate and transfer SUMO to target proteins (13,76,77). Here, we show that DNR induces a rapid and massive *de*SUMOylation of chromatin-bound proteins, in particular at active promoters and enhancers were SUMOylated proteins are highly enriched. As this occurs before massive *de*SUMOylation of other cellular proteins becomes detectable, this indicates that DNR-induced protein *de*SUMOylation is not random in the cell. It suggests it is kinetically and spatially ordered by mechanisms that remain to be characterized (also see below). It is however worth noting that, although DNR-induced *de*SUMOylation affects most genomic regions where SUMO-bound proteins were found in non-treated cells, new genomic regions, mostly intergenic, gain SUMOylation. As SUMO isoforms are limiting, DNR-induced deSUMOylation at promoters and enhancers could enhance the pool of unconjugated SUMO and favor the SUMOylation of other chromatin-bound proteins, such as Topoisomerase 2 and centromeric proteins (CENP-C and CENP-B), which we found up-SUMOylated upon DNR-treatment (Figure 4A). This might be also true for Ara-C, which also leads to a redistribution of SUMOylated proteins on the chromatin. However, contrarily to DNR, SUMOylated proteins are maintained at promoters and enhancers upon Ara-C treatment.

To address if DNR-induced *de*SUMOylation has a role in DNR-induced gene expression alterations, we performed RNA-Seq in cells treated with DNR and the SUMOylation inhibitor ML-792. As DNR induces fast chromatin protein *de*SUMOylation, we first asked whether inhibition of SUMOylation alone could reproduce its effect on gene expression. This was not the case as ML-792 had very small effects on gene expression (only 18 genes up-regulated and 3 down-regulated) after 3 h or treatment. This suggests that the inhibition of SUMOylation induced by DNR is not, on its own, responsible for the fast and broad transcriptome changes. This observation is consistent with the initial report on ML-792 showing that only a few genes are activated in cultured cells by this inhibitor, even after longer treatments (61). We therefore wondered whether protein *de*SUMOylation would have a role in the regulation of gene expression only in the presence of DNR. To this aim, we used ML-792 in combination with DNR, to accelerate and strengthen the *de*SUMOylation induced by this drug. Although ML-792 had little effect on the nature and the number of the genes up- or down-regulated upon DNR, it limited their up- or down-regulation. Indeed, most gene signatures enriched in DNR-treated cells were no longer enriched upon inhibition of SUMOylation. This suggested that acute *de*SUMOylation counteracts DNR ability to activate or repress gene expression. We however do not exclude that long-term and/or moderate hypo-SUMOylation could have a different effect on gene expression.

Our proteomic-based study of the HL-60 cell SUMOylome characterized the proteins that are *de*SUMOylated in response to DNR. Out of the 900 SUMOylated proteins identified in mock-treated cells, only 100 were significantly *de*SUMOylated after 2 h of DNR treatment. Consistent with the massive loss of SUMO-2/3 observed by ChIP-seq at promoters and enhancers at the same time point, most of these *de*SUMOylated proteins are transcription factors and co-regulators. This suggests that early DNR-induced *de*SUMOylation is spatially regulated and preferentially concerns proteins bound to specific chromatin regions, many of them probably being engaged in the same protein complexes. SUMOylation is indeed known to stabilize transcriptional complexes at gene regulatory regions to maintain transcription (16). For example, SUMOylation stabilizes transcription factor complexes involved in the expression of somatic transcriptional programs in MEFs (25,78). Massive increase in the SUMOylation of chromatin-bound protein upon heat-shock is also required to stabilize protein complex on gene regulatory regions to maintain their transcription (79). In both cases, protein complexes are likely modified following a process called ‘group SUMOylation’ (80). According to this concept, SUMO can control the activity of protein complexes regardless of the modified protein, or the precise sites that are SUMOylated on these proteins. DNR-induced *de*SUMOylation could loosen interactions within transcription-regulating complexes binding at the promoters and/or enhancers of the genes affected by DNR, thus limiting the transcription-promoting effect of DNR. If fast DNR-induced *de*SUMOylation at precise chromatin sites is most probably partly explained by local inhibition of chromatin-bound E1 and E2 SUMOylation enzymes, it might also involve faster deconjugation of SUMO by *de*SUMOylases at these same places. For example, SENP6 was reported to *de*SUMOylate CTCF (81), one of the proteins we found *de*SUMOylated by the DNR treatment. CTCF is a multifunctional protein involved in both the regulation of chromatin 3D architecture and the control of gene expression (82). It interacts with the cohesin complex (composed of SMC1, SMC3, RAD21 and SA1/2 proteins) and is involved in the formation of diverse chromatin regulatory loops (83). Depending on the situation, such loops can activate transcription by bringing enhancers and promoters in close proximity or repress it by limiting the access of transcriptional machineries or regulators to gene promoters (82). CTCF is SUMOylated (63,65), its SUMOylation being decreased by various stresses including hypoxia and oxidative stress (64). Further links between SUMO and CTCF were described on chromatin. First, the CTCF-binding consensus sequences was found enriched at genomic loci bound by SUMOylated proteins, in particular at promoters of inactive genes (84). Second, heat shock was shown to induce a transient depletion of SUMOylated proteins from CTCF-bound sites in intergenic regions and their relocation at promoters of transcribed genes (24). Third, SUMOylated proteins were found enriched at CTCF-bound sites in Drosophila and associated to enhancer blocking (85). Along this line, we found that the CTCF-binding site is the most enriched motif in SUMO-2/3 bound chromatin regions in AML cells and CUT&RUN experiments with CTCF antibodies confirmed that the co-binding of CTCF and SUMO is highly enriched at promoters and enhancers compared to intergenic regions. Moreover, the identification of CTCF as one of the proteins rapidly *de*SUMOylated upon DNR treatment, suggests that DNR-induced hypoSUMOylation of CTCF and probably of other still-to-be-identified proteins present at CTCF-bound sites could regulate the expression of specific genes through chromatin looping alteration. Hence, although we only identified four genes bound by CTCF and SUMO in their promoter and whose DNR-induced up- or down-regulation was blunted by ML-792 (more than 2-fold), we decided to explore this hypothesis. We focused on the *NFKB2* gene for several reasons: (i) it is one of the top-DNR-induced gene in HL-60 cells, (ii) its induction by DNR is reduced in the presence of ML-792 in HL-60 cells as well as in primary AML samples and (iii) its promoter region is both bound by CTCF and marked by SUMO and (iv) it plays important roles in the control of both cell survival and inflammation/immunity (66), two of the main gene categories rapidly affected by the DNR treatment. Our 4C experiments revealed that *NFKB2* promoter preferentially contacts four distal regions located up to 200 kb upstream (2 regions) and downstream (2 regions) of the *NFKB2* gene, all within the *NFKB2* containing TAD and bound by CTCF in HL-60 cells. Although DNR did not markedly alter the overall architecture of the *NFKB2* locus, it induced the loss of an interaction between *NFKB2* promoter and a region devoid of active histone marks (region IV) and the appearance of a new interaction with a candidate enhancer (region V). This probably reflects the loss of a transcription-repressive loop and the acquisition of a transcription-stimulating one. Consistent with its limited effects on gene expression, the sole inhibition of SUMOylation by the ML-792 inhibitor alone did not affect the overall structure of the *NFKB2* locus. This indicated that SUMOylation *per se* is not required for maintenance of the chromatin loops forming between the *NFKB2* promoter and the above-mentioned interacting regions (at least for the duration of the experiment). However, when used with DNR to accelerate and amplify DNR-induced *de*SUMOylation, ML-792 prevented the DNR-induced interaction between *NFKB2* promoter and the candidate enhancer located in region V. Instead, a new interaction with a region located beyond the TAD border (region VI) was induced. This switch might prevent full activation of *NFKB2* gene. Altogether, this suggests that *de*SUMOylation can attenuate the transcriptional effects of DNR by controlling chromatin 3D structure, at least on the *NFKB2* locus. Rapid and massive changes in the SUMO proteome associated to transcriptome alterations have already been observed in response to various external cues, including heat shock (24,79), oxidative stress (28,77) and genotoxics such as MMS (86). Our herein data suggest that such SUMO-dependent switches might control transcriptome changes at least in part by affecting chromatin 3D architecture or dynamics. This is all the more to be considered that inducible genes have been reported to be more enriched in CTCF-controlled chromatin loops than housekeeping ones (87,88). Future work will therefore have to elucidate whether SUMO serves as a platform, especially at CTCF-bound sites, to recruit proteins involved in chromatin remodeling or structuration and how SUMOylation/*de*SUMOylation cycles at these places contributes to transcriptional changes linked to alteration of 3D chromatin organization.

## Supplementary Material

gkad581_Supplemental_Files

## Data Availability

Microarray data were deposited on Arrayexpress with accession number E-MATB-4895. ChIPSeq, RNA-Seq, 4C and CUT&RUN sequencing data were deposited on Gene Expression Omnibus with accession number GSE198986. Data can be visualized on the UCSC genome browser: https://genome.ucsc.edu/s/MathiasBoul/Boulanger%20et%20al%20%2D%20HL60%20datasets. The mass spectrometry proteomics data have been deposited to the ProteomeXchange Consortium via the PRIDE partner repository with the dataset identifier PXD032956.
