# Supplementary material for: DeSUMOylation of chromatin-bound proteins limits the rapid transcriptional reprogramming induced by daunorubicin in acute myeloid leukemias
Source: Nucleic Acids Res. 2023 Jul 18;51(16):8413–33. doi: 10.1093/nar/gkad581 (PMC10484680; doi:10.1093/nar/gkad581)
Supplement: gkad581_Supplemental_Files [file gkad581_supplemental_files.zip › 2023-04-28 Supplementary Figures.pdf]

Supplementary Figures

Supplementary Figure 1

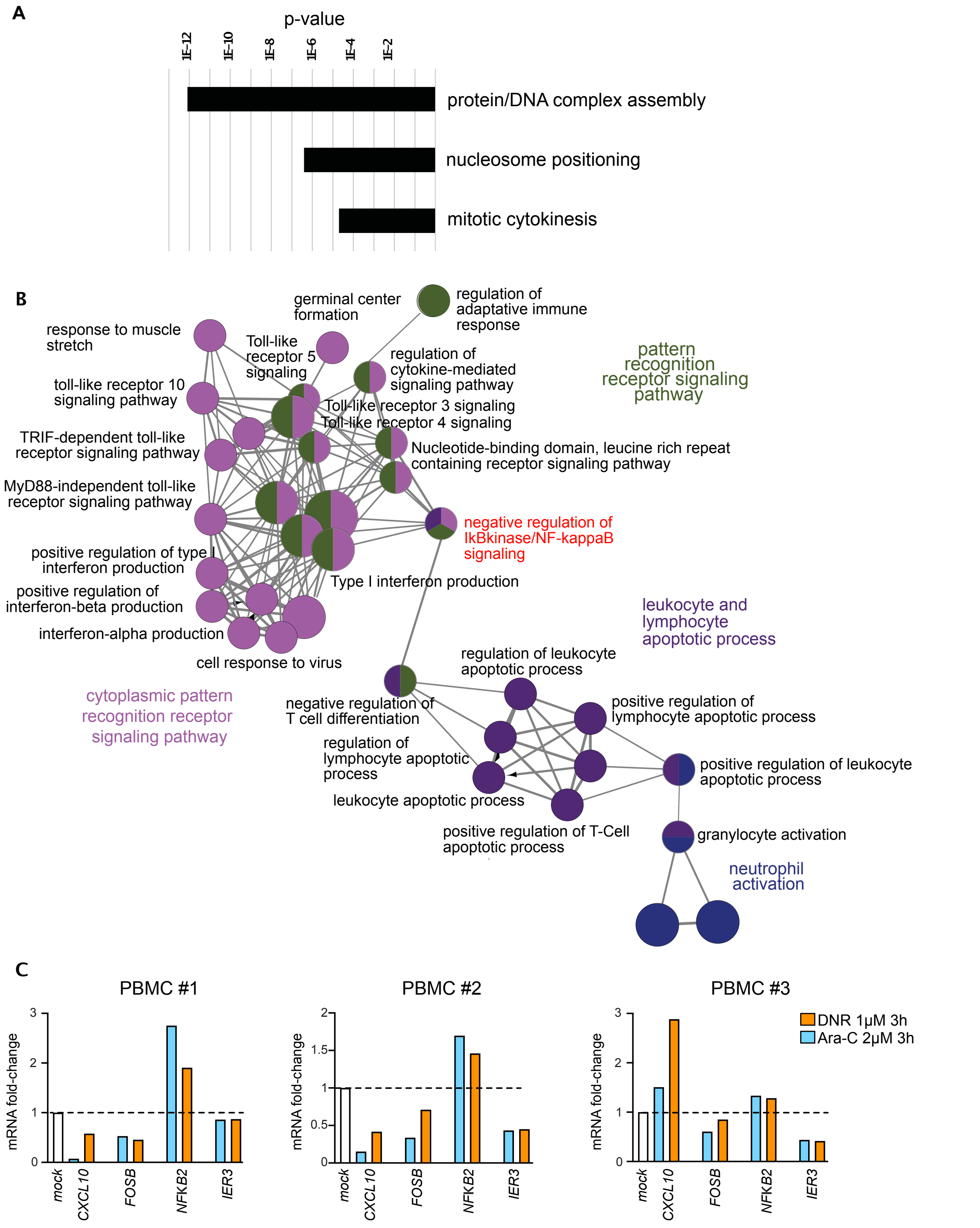

Supplementary Figure 1: Chemotherapeutic drugs induce a rapid transcriptional

reprogramming in AML cells. *A: Ontology analysis of the genes down-regulated (<-2 fold, FDR<0.05) by DNR and Ara-C.* Ontologies were obtained using the ClueGo plugin in the Cytoscape application. *B: String network of genes up-regulated by DNR.* The network was obtained with the ClueGo plugin in the Cytoscape application using all genes up-regulated (>2 fold, FDR<0.05) by DNR. C: PBMCs from 3 healthy donors were treated *in vitro* with 1 µM DNR or 2 µM Ara-C for 3 hours. The levels of the indicated mRNAs were measured by RT-qPCR, normalized to *GAPDH* levels and expressed as fold increase to mock-treated cells.

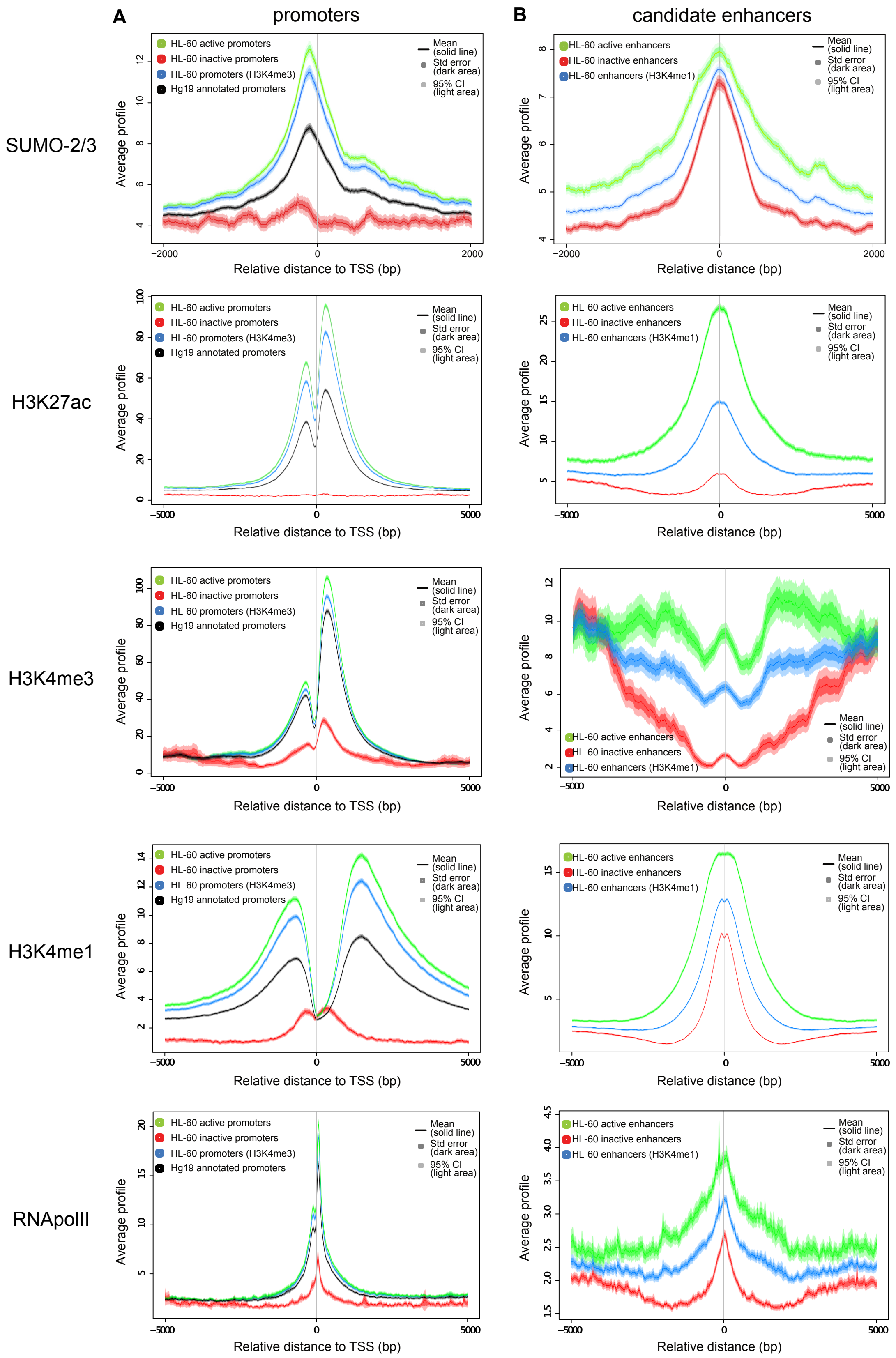

Supplementary Figure 2: SUMO-2/3 conjugated proteins are enriched on enhancers and active promoters. *A, B: Metaprofiles of the SUMO-2/3* ChIP-seq signal on promoters (A) or enhancers (B) depending of the characteristics of *cis*-regulatory element. The promoters and

enhancers as well as their level of activity were defined using publicly available histone marks ChIP-seq data (H3K27ac, H3K4me3 and H3K4me1) and RNApolII for HL-60 cells and NCBI refseq data.

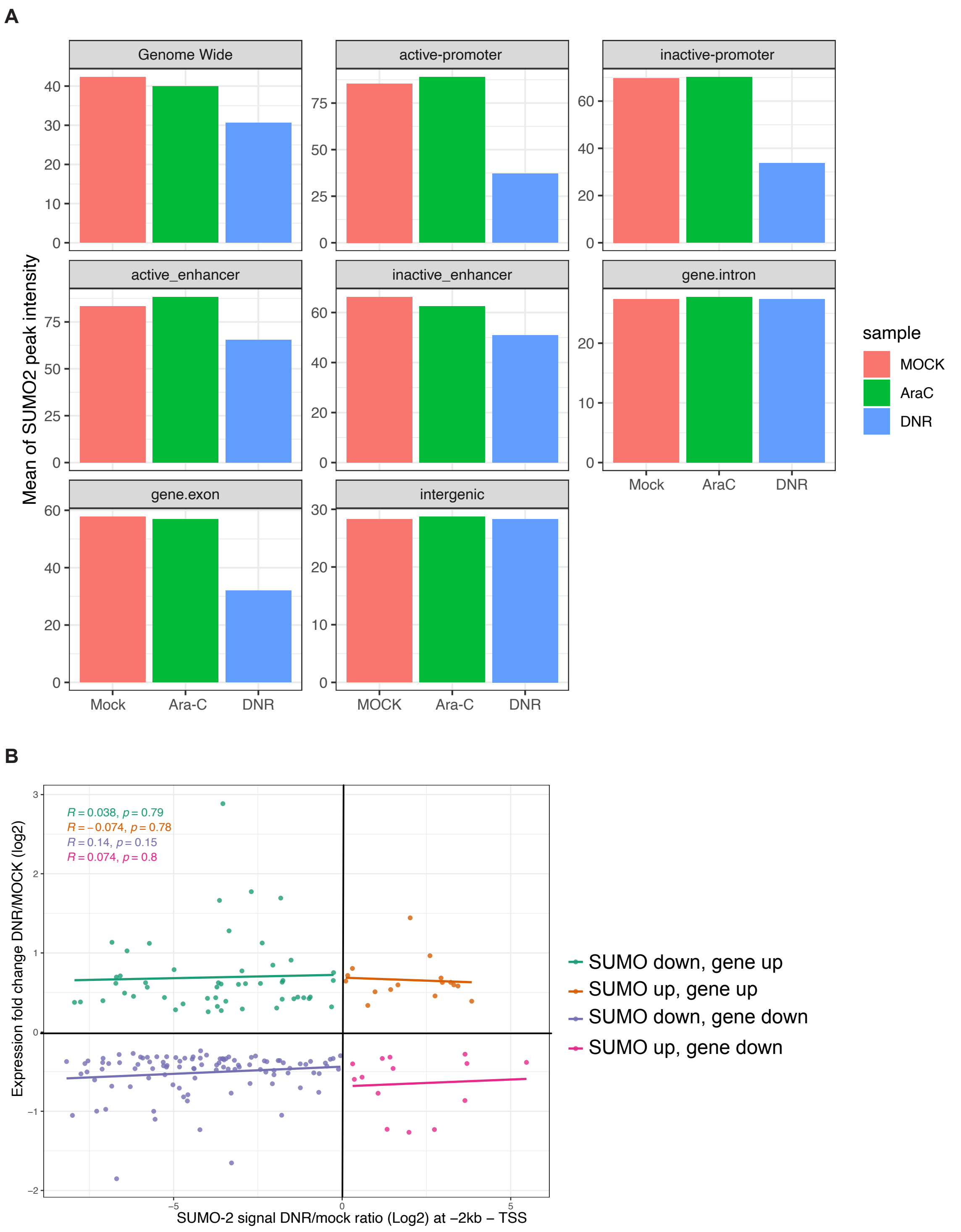

Supplementary Figure 3: Effect of DNR and Ara-C on chromatin regions SUMOylation. *A: Histogram representing the mean SUMO-2/3 ChIP-Seq peak intensity* in mock-, DNR- or Ara-C treated cells depending on the different genomic regions. For each condition, the level of SUMO-2/3 signal has been analyzed at each identified peak. The promoters and enhancers as well as their level of activity were defined using publicly available histone marks ChIP-seq data (H3K27ac, H3K4me3 and H3K4me1) and RNApolII for HL-60 cells and NCBI refseq data. *B: Changes in SUMO-2/3 at gene promoters during DNR treatment is not correlated with DNR-induced gene regulatio*n. Scatter plot of gene expression fold change upon DNR treatment as a function of the changes in SUMO2/3 signal at promoters defined as the region the 2kb upstream the TSS. The linear regression for each group is shown by the coloured lines and respective parameters are indicated (top left) corner. The genes with no significative changes in expression upon DNR treatment nor SUMO2/3 variations within their promoter are masked.

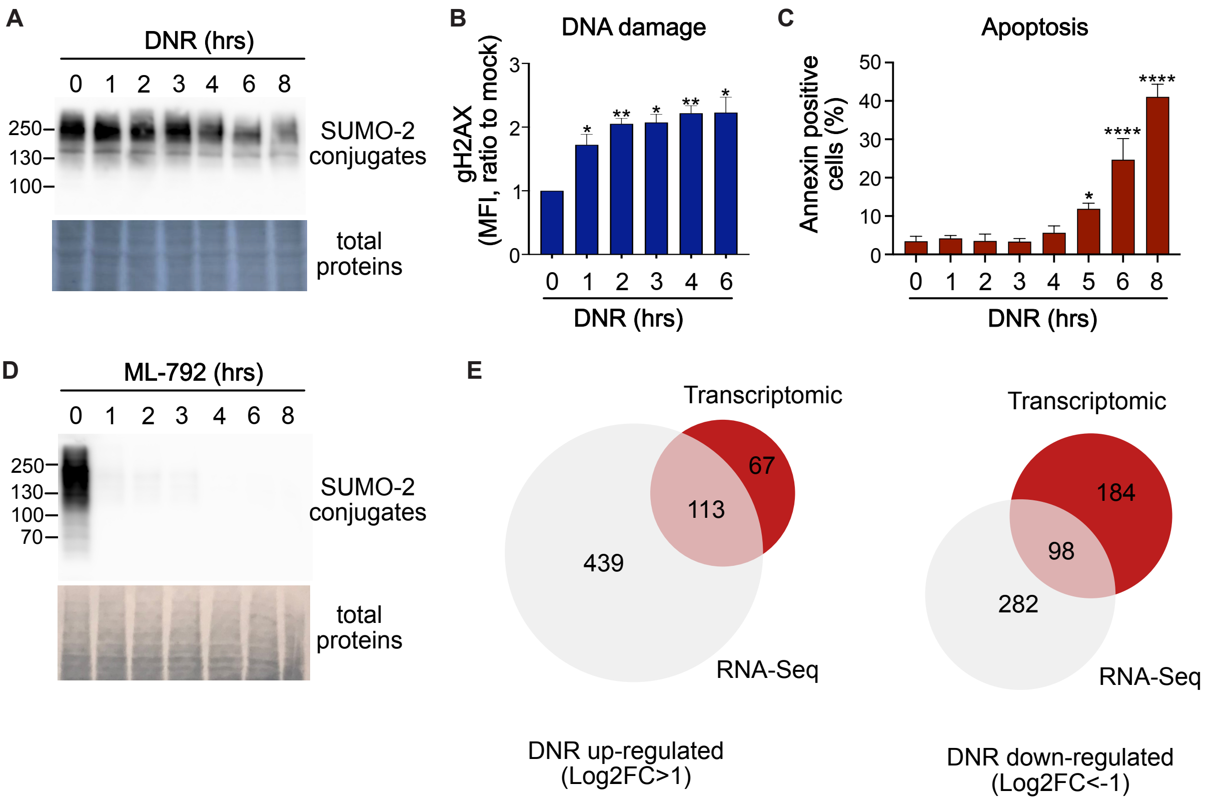

Supplementary Figure 4: DNR induces deSUMOylation and transcriptional reprogramming in HL-60 cells. *A: Kinetic of DNR-induced deSUMOylation.* HL-60 cells were treated with DNR (1 µM) for the indicated times. Total cell extracts were loaded on SDS-PAGE and immunoblotted for SUMO-2/3 (upper panel) and stained with amidoblack (lower panel). *B-C: DNA damage and apoptosis induced by DNR.* HL-60 cells were treated with DNR (1 µM) for the indicated times and labelled with FITC-coupled γH2AX antibody (B) or FITC-

Annexin V and analyzed by flow cytometry (n=3, RM-One Way Anova). *D*: *Kinetic of ML-792-induced deSUMOylation.* HL-60 cells were treated with ML-792 (0.5 µM) for the indicated times. Total cell extracts were loaded on SDS-PAGE and immunoblotted for SUMO-2/3 (upper panel) and stained with amidoblack (lower panel). *E: Venn diagram* for the genes found up-regulated (left panel) or down-regulated (right panel) in the transcriptomic and RNA-Seq approaches.

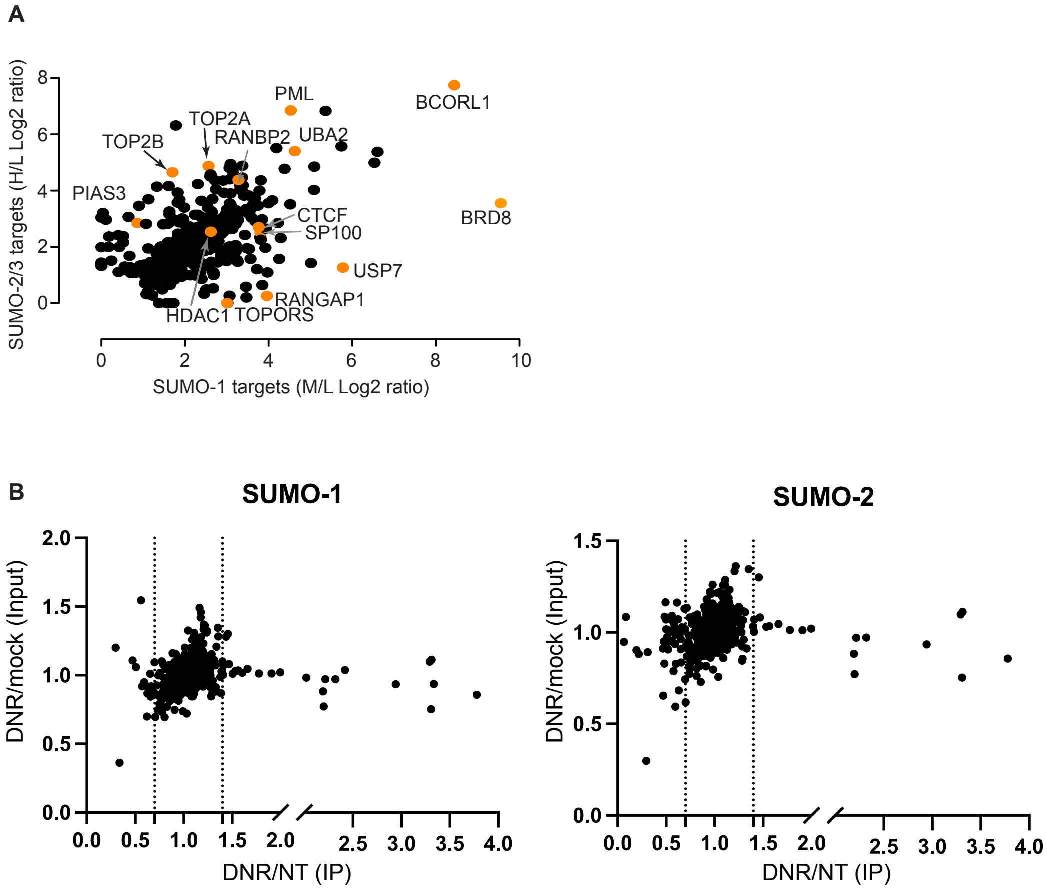

Supplementary Figure 5: Identification of endogenously SUMOylated proteins in HL-60 cells. *A: Endogenously SUMOylated proteins in HL-60 cells.* SUMOylated proteins were immunoprecipitated from triple SILAC-labeled HL-60 cells with control (light condition), anti SUMO-1 (medium condition) or anti SUMO-2/3 (heavy condition) antibodies and identified by mass spectrometry. Log2-transformed ratios between SUMO-1 or SUMO-2/3 and control IP are represented. Only proteins with more than 2 peptides identified and a Log2 ratio>1 (SUMO-1/control or SUMO-2/control) were selected. The orange dots correspond to selected known SUMO substrates. *B: DNR treatment does not affect SUMOylated proteins abundance.* SILAC ratio between DNR- and mock-treated HL-60 cells in the total cell extract (input sample) and in the SUMO-1 (left panel) or SUMO-2/3 (right panel) immunoprecipitation.

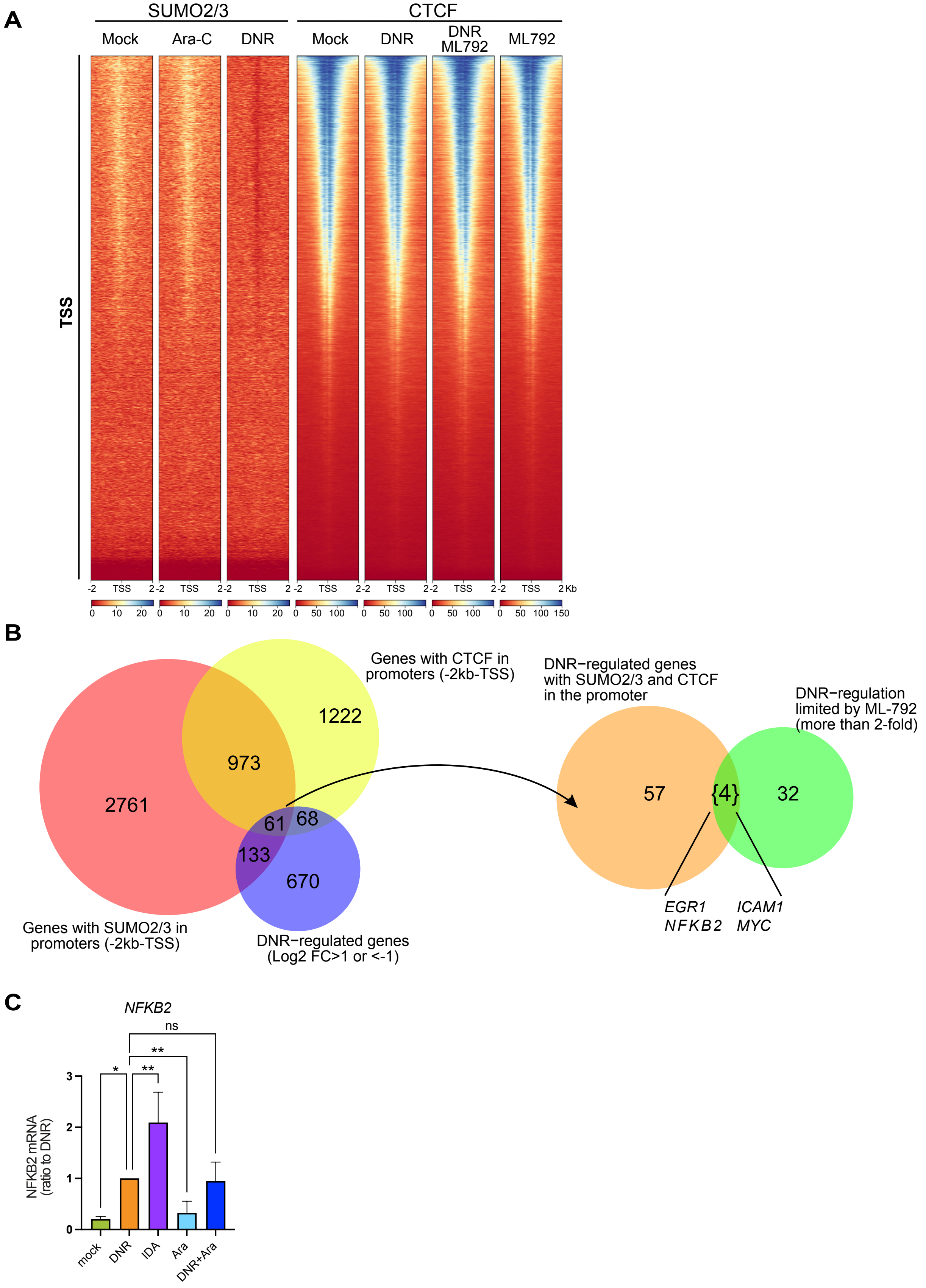

Supplementary Figure 6: CTCF and SUMO colocalize at gene promoters. (A) Heat-map

for the distribution of SUMO-2 (ChIP-Seq) and CTCF (CUT&RUN) at TSS in HL-60 cells. The ranking was made according to SUMO-2/3 signal. *B: identification of genes potentially regulated by SUMO and CTCF.* Left panel*:* Venn Diagram showing the intersection between the genes bound by SUMO-2/3 and CTCF in their promoter regions (-2kb- TSS) and whose expression is up- or down-regulated ≥2 fold upon DNR treatment. Right panel: Venn diagram displaying the intersection between the 61 genes bound by both SUMO and CTCF and regulated by DNR with the list of 36 genes whose DNR-induced regulation is affected ≥2-fold by the inhibition of SUMOylation. *C: Induction of NFKB2 gene by chemotherapeutic treatments.* HL-60 were treated for 3 hrs with DNR (1 µM), IDA (1 µM), Ara-C (2 µM) or DNR+Ara-C. The levels of *NFKB2* mRNAs were measured by RT-qPCR, normalized to *GAPDH* and expressed as ratio to DNR-treated cells (n=4, Ordinary One-Way Anova).

Supplementary Table 1: Transcriptomic analysis of DNR and Ara-C regulated genes.

Supplementary Table 2: Characteristics of patient samples used in this study

Supplementary Table 3: RNA-Seq analysis of the genes regulated by DNR +/- ML-792

Supplementary Table 4: GSEA analysis of the RNA-Seq analysis

Supplementary Table 5: SILAC mass spectrometry identification of SUMOylated proteins.

Supplementary Table 6: HOMER motif search at SUMO-2/3 bound sites on chromatin
